# Wittichenite semiconductor of Cu_3_BiS_3_ films for efficient hydrogen evolution from solar driven photoelectrochemical water splitting

**DOI:** 10.1038/s41467-021-24060-5

**Published:** 2021-06-18

**Authors:** Dingwang Huang, Lintao Li, Kang Wang, Yan Li, Kuang Feng, Feng Jiang

**Affiliations:** 1 Institute of Semiconductor Science and Technology, South China Normal University, Guangzhou, P. R. China; 2 SCNU Qingyuan Institute of Science and Technology Innovation Co., Ltd., Qingyuan, China; 3 Guangdong Provincial Engineering Technology Research Center for Low Carbon and Advanced Energy Materials, South China Normal University, Guangzhou, China

**Keywords:** Photocatalysis, Artificial photosynthesis, Photocatalysis

## Abstract

A highly efficient, low-cost and environmentally friendly photocathode with long-term stability is the goal of practical solar hydrogen evolution applications. Here, we found that the Cu_3_BiS_3_ film-based photocathode meets the abovementioned requirements. The Cu_3_BiS_3_-based photocathode presents a remarkable onset potential over 0.9 V_RHE_ with excellent photoelectrochemical current densities (~7 mA/cm^2^ under 0 V_RHE_) and appreciable 10-hour long-term stability in neutral water solutions. This high onset potential of the Cu_3_BiS_3_-based photocathode directly results in a good unbiased operating photocurrent of ~1.6 mA/cm^2^ assisted by the BiVO_4_ photoanode. A tandem device of Cu_3_BiS_3_-BiVO_4_ with an unbiased solar-to-hydrogen conversion efficiency of 2.04% is presented. This tandem device also presents high stability over 20 hours. Ultimately, a 5 × 5 cm^2^ large Cu_3_BiS_3_-BiVO_4_ tandem device module is fabricated for standalone overall solar water splitting with a long-term stability of 60 hours.

## Abstract (web summary)

A highly efficient, stable, low-cost and environmentally friendly photocathode is the goal of practical solar hydrogen evolution applications. Here, authors report a Cu_3_BiS_3_-based photocathode and Cu_3_BiS_3_-BiVO_4_ tandem cell for unbiased overall solar water splitting with a STH efficiency over 2%.

## Introduction

Solar water splitting using sunlight irradiation has been widely believed to be a clean way to produce hydrogen energy without CO_2_ emission^1–4^. As an appreciable solar energy utilization/conversion mode, photoelectrochemical (PEC) solar water splitting has been extensively studied based on various semiconductors^5–7^. Since Honda and Fujishima found that TiO_2_ photoelectrodes showed promising PEC water splitting properties^8^, various semiconductor materials have been investigated to improve the solar-to-hydrogen (STH) conversion efficiency and device working stability^9^. The core of the PEC water splitting device is at the semiconductor/liquid interface junction, where minority carriers excited in the semiconductor are driven into the liquid by the electric field in the depletion region at the semiconductor/liquid interface^6^. For example, redox reactions, such as the reduction of H^+^ to H_2_, are driven at the interface between the P-type semiconductor and liquid. To date, many p-type semiconductors have been studied as photocathodes for PEC water splitting.

P-type semiconductors that are used on photovoltaic devices such as c-Si^10^, a-Si^11^, Cu(In,Ga)Se^12,13^, CuInS_2_^14^, Cu_2_ZnSnS_4_ (CZTS)^15,16^, CuSbS_2_^17^, Sb_2_Se_3_^18^ and CdTe^19^ are always suitable as the photocathode for PEC water splitting due to their high light absorption coefficient and suitable optical band gap. In recent years, Cu-chalcogenide compound semiconductors have attracted wide attention for PEC water splitting. Although Cu-chalcogenide photocathodes such as CuInS_2_^14^, CuGaSe_2_^20^, CuGa_3_Se_5_^21^, (Ag,Cu)GaSe_2_^22^, and Cu(In,Ga)Se_2_^12,13^, show excellent STH conversion efficiency, there are many difficulties in their large-scale production with regards to low throughput and material utilization because of the limited availability of elements (i.e., In, Ga and Se). Therefore, cost-effective and environmentally protective CZTS semiconductors are believed to be promising photocathode materials that have been investigated for ~10 years since the first report from Prof. Domen’s group in 2010^23^. During the last few years, many researchers, including us, have made efforts to improve the STH conversion efficiency and working stability^15,16,24–28^. Recently, we achieved a record applied bias photon-to-current efficiency (ABPE) over 2.7% and 3.5% with the highest onset potential of 0.7 V_RHE_ and over 10 h of PEC stability for the CZTS-based photocathode by various surface modifications^25,28^. However, the complex element consistency and numerous antisite/vacancy defects of CZTS compounds restrict their photovoltage. The record onset potential we achieved at ~0.7 V_RHE_ is still not appreciable for the further fabrication of efficient tandem cells with suitable photoanodes such as BiVO_4_ and Fe_2_O_3_^29,30^. Meanwhile, the required postsulfurization process of the CZTS film also restricted its large-scale industrial integration for solar hydrogen evolution^16,25,28^. Cu_2_O-based photocathodes have been widely investigated due to their high PEC photovoltage, which has reached as high as 1.2 V_RHE_, but their low stability is still a major problem^31,32^. Fortunately, ternary I − V − VI compounds of Cu_3_BiS_3_ are believed to be promising chalcogenide compounds for photovoltaic devices due to their perfect optical band gap (*Eg* ≈ 1.4–1.7 eV), high absorption coefficient (>10^5^ cm^−1^) compared to those of CuInSe_2_ and Cu_2_ZnSnS_4_, and p-type conductivity with a carrier concentration of ~2 × 10^16^ cm^−3^ ^33–35^. Cu_3_BiS_3_, naturally occurring in the “Wittichenite” mineral form, is stable for a broad range of processing temperatures^36^. Moreover, it is made of inexpensive, nontoxic, and earth-abundant elements, and we found that the Cu_3_BiS_3_ film can be easily produced on a large scale by any suitable low-cost approach not under vacuum, such as printing and spraying^34,37^. Recently, Cu_3_BiS_3_ has attracted much attention, and remarkable progress has been made by many groups using different fabrication approaches and theoretical studies^38–40^.

However, the previously reported photovoltaic performances and PEC water splitting efficiencies of Cu_3_BiS_3_-based solar cells or photocathode devices are still not significant^35,37–39^. The best conversion efficiency achieved by a Cu_3_BiS_3_-based solar cell is 0.17% (0.11 cm^2^ active area)^37^, and the best reported Cu_3_BiS_3_-based photocathode only presented a 0.1 mA/cm^2^ photocurrent density at 0 V_RHE_ (*J*_*0*_), with a 0.65 V_RHE_ onset potential value (*Voc*)^38^. Moreover, the reported devices are all on the lab scale, and their stabilities are all not appreciable (<60 min)^37–39^. The reported low efficiency and poor PEC performance of Cu_3_BiS_3_-based photoelectrodes are far from their theoretical limit considering their perfect optical band gap value of ~1.7 eV for solar water splitting, and the low working stability itself restricts their further industrial utilization^38,39^. The low solar-to -hydrogen conversion efficiency should be caused by the low crystallinity of the material, poor surface/interface conditions and obvious internal defects^33,38^. In this work, we presented a world record ABPE of 1.7% for our Cu_3_BiS_3_-based photocathode for solar water splitting, and the photocurrent density (*J*_*0*_) of ~7 mA/cm^2^, onset potential (*Voc*) of 0.9 V_RHE_ and ABPE of 1.7%. The obtained photocurrent density (*J*_*0*_) of ~7 mA/cm^2^ are almost 70 times higher than their previously reported highest values of *J*_*0*_ (0.1 mA/cm^2^)^38^. In addition, due to the high onset potential of the Cu_3_BiS_3_-based photocathode, we first fabricated a tandem cell of Cu_3_BiS_3_-BiVO_4_ for unbiased solar water splitting, and an unassisted bright STH conversion efficiency over 2% was also achieved. More importantly, a large-scale Cu_3_BiS_3_-based single photocathode over 5 × 5 cm^2^ was also fabricated. Finally, with the help of the BiVO_4_ photoanode, a large 5 × 5 cm^2^ Cu_3_BiS_3_-BiVO_4_ tandem cell was fabricated. We were excited to find that the large-scale Cu_3_BiS_3_-BiVO_4_ tandem cell showed long-term working stability, and the device working stability was found to be very high; the solar water splitting photocurrents were not degraded over 60 h (70% of their initial photocurrent density was maintained).

This is the first report of an efficient and large-sized Cu_3_BiS_3_-based photocathode and Cu_3_BiS_3_-BiVO_4_ tandem cell for unbiased solar water splitting. The large device size, appreciable STH conversion efficiency and high device working stability that were presented in this work indicated the promising practical application potentials of the Cu_3_BiS_3_-based photoelectrode.

## Results

### Fabrication and optimization of the Cu_3_BiS_3_ thin films

In this work, Cu_3_BiS_3_ thin films were prepared by spray pyrolysis in one step. The detailed raw materials and process for the preparation of Cu_3_BiS_3_ films are given in the Supporting Information. We found that the quality of the Cu_3_BiS_3_ films was significantly influenced by the substrate temperature and Cu: Bi molar ratio of the precursor solution.

**Fig. 1:**
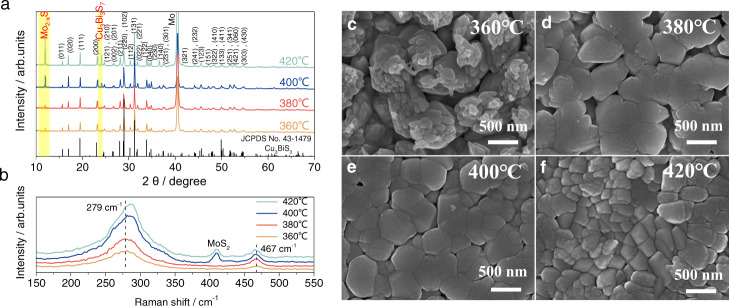
Structural characterization of the Cu_3_BiS_3_ thin films. (**a**) XRD patterns and (**b**) Raman spectra of the Cu_3_BiS_3_ films sprayed at various substrate temperatures. surface SEM images of the Cu_3_BiS_3_ films sprayed at various substrate temperatures of (**c**) 360 °C, (**d**) 380 °C, (**e**) 400 °C and (**f**) 420 °C. Source data are provided as a Source Data file.

Figure 1a shows the XRD patterns of the Cu_3_BiS_3_ films sprayed at various substrate temperatures (360 °C, 380 °C, 400 °C and 420 °C). It was found that the intensity of major diffraction peaks increased with increasing substrate temperature, indicating that the crystallinity of Cu_3_BiS_3_ films was improved with the substrate temperature. Nevertheless, some impurity phases, such as Mo_2-x_S and Cu_3_Bi_3_S_7,_ were also easily formed at high substrate temperatures (marked with a yellow background in Fig. 1a). Furthermore, the substrate temperature-dependent Raman spectrum of the Cu_3_BiS_3_ films is shown in Fig. 1b. We observed two major vibrational peaks at 279 cm^−1^ and 467 cm^−1^ from the Raman spectrum, which matched well with the reported Raman shifts of Cu_3_BiS_3_ films^33,38^. The half-widths of the vibrational peaks at ~279 cm^−1^ and 467 cm^−1^ decreased with increasing substrate temperature, indicating that the crystallinity increased with increasing substrate temperature. In addition, an extra Raman shift peak at ~410 cm^−1^ (corresponding to the MoS_2_ Raman peak) appeared when the substrate temperature increased to above 400 °C. Notably, post-high-temperature sulfurization is generally necessary for the further crystal growth of multimetal sulfide materials^13–17^. Nevertheless, the one-step sprayed Cu_3_BiS_3_ films in this work possessed good crystalline quality and pure phases with no obvious secondary phase or impurity phase, indicating that no postannealing process was required, which was instrumental in scalability and practical applications for Cu_3_BiS_3_-based photocathodes.

The microstructural morphologies of the Cu_3_BiS_3_ films sprayed at various substrate temperatures (360 °C, 380 °C, 400 °C and 420 °C) are shown in Fig. 1c–f. The films sprayed at a relatively low substrate temperature of 360 °C were not compact, various small crystals composed the Cu_3_BiS_3_ films, and obvious fluctuations with large gaps were observed from their surface morphology (Fig. 1c). When we increased the substrate temperature to 380 °C, the Cu_3_BiS_3_ crystal size significantly increased to ~1000 nm, and the films were observed to be compact and flat (Fig. 1d). Although the Cu_3_BiS_3_ films sprayed at 400 °C were dense and smooth, the crystal size of Cu_3_BiS_3_ was slightly decreased (Fig. 1e). Moreover, when the substrate temperature increased to 420 °C, the size of the Cu_3_BiS_3_ crystals obviously decreased to ~300–500 nm (Fig. 1f).

**Fig. 2:**
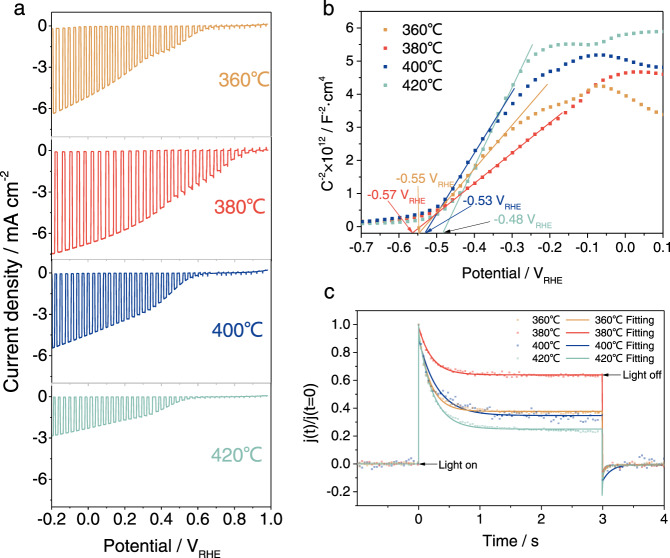
Performance and charge transfer dynamics characterization of the Cu_3_BiS_3_-based photocathode sprayed at various substrate temperatures. (**a**) Chopped photocurrent density-potential curves, (**b**) corresponding Mott–Schottky curves and (**c**) corresponding transit photocurrent spectra of the Cu_3_BiS_3_-based photocathodes sprayed at various substrate temperatures (360 °C, 380 °C, 400 °C and 420 °C). Source data are provided as a Source Data file.

Figure 2a shows the PEC performance of the Cu_3_BiS_3_-based photocathodes sprayed at various substrate temperatures determined by LSV under chopped sunlight irradiation. The photocathode based on the Cu_3_BiS_3_ film sprayed at 380 °C exhibited the highest photocurrent density (~7 mA/cm^2^), onset potential (~0.9 V_RHE_) and fill factor. Meanwhile, the statistical plots shown in Fig. S1 also persuasively suggested this trend. The flat band potential (*V*_*fb*_) of the Cu_3_BiS_3_-based photocathodes sprayed at various substrate temperatures can be determined from the intersecting points of the Mott–Schottky curves and *X* axis (shown in Fig. 2b)^41^. The photocathodes based on the Cu_3_BiS_3_ films sprayed at 360 °C, 380 °C, 400 °C and 420 °C exhibited V_fb_ values of −0.55 V_RHE_, −0.57 V_RHE_, −0.53 V_RHE_ and −0.48 V_RHE_, respectively. The most negative Vfb of −0.57 V_RHE_ (corresponding to the photocathode based on the Cu_3_BiS_3_ film sprayed at 380 °C) implied the highest energy level of photogenerated electrons, indicating that it was easier for photogenerated electrons to transfer from the electrode into the electrolyte and participate in the hydrogen evolution reaction^41^.

Figure 2c shows the transient photocurrent decay of the photocathodes based on the Cu_3_BiS_3_ films sprayed at various substrate temperatures (360 °C, 380 °C, 400 °C and 420 °C). We found that the photocathode based on the Cu_3_BiS_3_ film sprayed at 380 °C exhibited a smaller photocurrent spike and wider saturated photocurrent region, illustrating less charge carrier recombination and efficient photogenerated electron transfer within the electrode^28^. In summary, the photocathode based on the Cu_3_BiS_3_ film sprayed at 380 °C invariably presented excellent charge-transfer kinetics, which may be attributed to the fact that the Cu_3_BiS_3_ film sprayed at the 380 °C substrate temperature possessed fewer spurious phases, fewer bulk defects and higher crystallinity. As shown in Fig. S2, electrochemical impedance spectroscopy (EIS) measurements were undertaken to further identify the charge-transfer kinetics mechanism of the Cu_3_BiS_3_-based photocathodes sprayed at various substrate temperature. The EIS measurements were carried out in phosphate buffer (pH = 6.5) under AM 1.5 G illumination biased at 0.3 V_RHE_ with a frequency sweep from 100 kHz to 0.1 Hz. An equivalent circuit model (shown in the inset of Fig. S2) consisting of two serially connected resistor–capacitor blocks with a resistance (*R*) and a constant phase element was employed to fit the EIS results^42^. The fitted resistance parameters are shown in Table S1, in which *R*_*s*_ is mainly composed of the sheet resistance of the contact and external wire. *R*_*1*_ is generally influenced by the transport of charge carriers inside the electrode, and *R*_*2*_ represents the impedance at the electrode/electrolyte interface^33–40^. We found that the samples sprayed at 380 °C showed a significantly lower bulk resistance and lower interfacial/bulk recombination ratio of photoexcited carriers due to the compact film structure and appreciate crystalline quality of the Cu_3_BiS_3_ film (380 °C), as shown in Fig. 1d. The Cu_3_BiS_3_ films we studied below were all sprayed at 380 °C substrate temperature.

The effects of the Cu:Bi molar ratio of the precursor solution on the crystallinity and purity of the sprayed Cu_3_BiS_3_ films were also systematically investigated in this work. Figure S3 shows a comparison of the X-ray diffraction (XRD) patterns of Cu_3_BiS_3_ films sprayed from precursor solutions with various Cu: Bi molar ratios. Clearly, all of the observed diffraction peaks of the Cu_3_BiS_3_ film sprayed from the precursor solution with a Cu:Bi = 3:1 ratio corresponded well to the reference (JCPDS NO. 43-1479)^33–40^. Nevertheless, we observed obvious secondary phases and impurity phases, such as Cu_2-x_S and Cu_3_Bi_3_S_7_ (marked with a yellow background in Fig. S3), from the diffraction peaks of the Cu_3_BiS_3_ films sprayed from the precursor solution with Cu:Bi ratios of 1:1, 2:1, 4:1 and 5:1. Meanwhile, the high intensity of sharp diffraction characteristic peaks of the Cu_3_BiS_3_ film (with a Cu:Bi = 3:1 ratio) indicated its good crystallinity, which was preferred for high-performance water splitting device fabrication.

Furthermore, the PEC performance of the Cu_3_BiS_3_-based photocathode as a function of various Cu:Bi molar ratios was also discussed. As we expected, the Cu_3_BiS_3_-based photocathode with a Cu:Bi = 3:1 ratio exhibited the highest photocurrent density and onset potential, as shown in Fig. S4a. We repeated the experiments many times and found the real trend, as shown in Fig. S4b. Relatively, the samples with an excess of Cu tended to show a higher PEC performance than Cu-deficient samples, but this trend was reversed when the molar ratio of Cu:Bi exceeded 3:1. Therefore, the stoichiometric samples possessed the highest PEC performance (including photocurrent density, onset potential and ABPE). The EIS spectra (Fig. S5) and the fitted resistance parameters results (Table S2) of these Cu_3_BiS_3_ photocathodes also indicated that the stoichiometric samples exhibited the smallest values of* R*_*1*_*´* and *R*_*2*_*´*, which suggested less charge recombination inside of the electrode and better charge transfer at the electrode/electrolyte interface^42^. The EIS analyses were consistent with the results of linear sweep voltammetry (LSV) measurements (Fig. S4a). As a result, the optimal Cu:Bi ratio of the precursor solution for the sprayed Cu_3_BiS_3_ films was 3:1.

On the basis of these studies, the Cu_3_BiS_3_ film sprayed at a 380 °C substrate temperature and with a Cu:Bi = 3:1 molar ratio precursor solution was selected as the absorption layer for the Cu_3_BiS_3_ photocathode. In this context, transmission electron microscopy (TEM) of the optimum Cu_3_BiS_3_ film (Fig. S6a) further confirmed the large Cu_3_BiS_3_ crystalline size of ~1000 nm. The STEM-EDX elemental mappings of Cu, Bi and S in Fig. S6 b–d clearly showed that the elements were homogeneously distributed in the grains. In addition, the high-resolution transmission electron microscopy image of the as-prepared Cu_3_BiS_3_ crystals (Fig. S6e) indicated that the measured interplanar distance of 2.8 Å and 3.0 Å corresponds to the (131) and (102) plane of the Wittichenite Cu_3_BiS_3_ (PDF No. 43-1479). The typical selected area electron diffraction pattern (Fig. S6f) indicated that our Cu_3_BiS_3_ film possessed a good crystalline quality.

### PEC performance of the Cu_3_BiS_3_-based photocathode

As we have found in the CZTS-based photocathode, surface coverage of the CdS buffer layer is an effective way to enhance the PEC performance due to the p–n junction formed at the interface of CZTS/CdS^16,25,26,28^, and further deposition of the TiO_2_ protective layer under CdS would enhance the PEC stability^43^. In this work, a TiO_2_/CdS overlayer and a Pt particle catalyst were also modified under the Cu_3_BiS_3_ absorption layer, and the detailed deposition processes of these modification layers are given in the Supporting Information. The microscopic cross-sectional structure of the finished Pt-TiO_2_/CdS/Cu_3_BiS_3_ electrode is shown in Fig. S7a, indicating that a very thin TiO_2_ (~50 nm)/CdS (~80 nm) double layer was modified onto the surface of the Cu_3_BiS_3_ layer (~1000 nm). The EDS mapping results (Fig. S7b–d) clearly show the interfaces between the multilayer structure of TiO_2_/CdS/Cu_3_BiS_3_, demonstrating the compact coverage or passivation of the Cu_3_BiS_3_ films by CdS and TiO_2_ over layers.

**Fig. 3:**
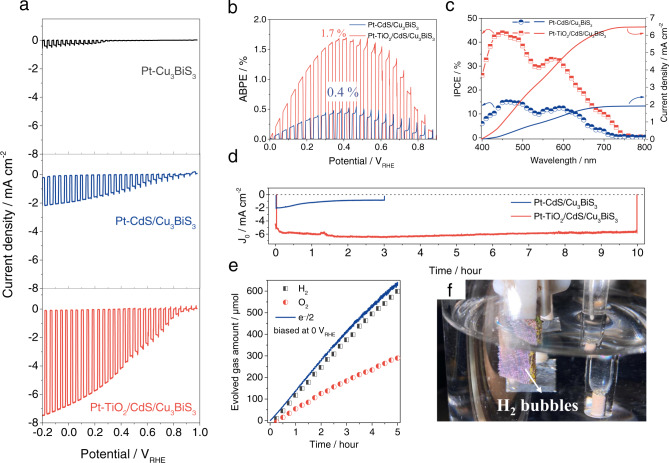
PEC performance of the Cu_3_BiS_3_-based photocathodes. (**a**) Chopped photocurrent density-potential curves of the Pt-Cu_3_BiS_3_, Pt-CdS/Cu_3_BiS_3_ and Pt-TiO_2_/CdS/Cu_3_BiS_3_ photocathodes. (**b**) ABPE curves, (**c**) IPCE spectra and (**d**) photocurrent density-time curves of the Pt-CdS/Cu_3_BiS_3_ and Pt-TiO_2_/CdS/Cu_3_BiS_3_ photocathodes. (**e**) Hydrogen and oxygen evolution amounts of the Pt-TiO_2_/CdS/Cu_3_BiS_3_ photocathode with various illumination times and (**f**) photograph of the Pt-TiO_2_/CdS/Cu_3_BiS_3_ photocathode under working conditions. All measurements were carried out in a 0.2 mol/dm^3^ Na_2_HPO_4_/NaH_2_PO_4_ solution (pH = 6.5) under solar simulated AM 1.5 G light irradiation. Source data are provided as a Source Data file.

The PEC performances of the Pt-Cu_3_BiS_3_, Pt-CdS/Cu_3_BiS_3_ and Pt-TiO_2_/CdS/Cu_3_BiS_3_ photocathodes were investigated by LSV, as shown in Fig. 3a. Pt catalytic particles are necessary to provide active sites for surface reactions^43^. However, the Pt-Cu_3_BiS_3_ photocathode exhibited a poor photocurrent density that was even <1 mA/cm^2^. As a buffer layer, a chemical bath deposited (CBD) CdS layer was modified onto the Cu_3_BiS_3_ layer to enhance the PEC performance of the photocathodes, which was usually applied onto various absorber layers, such as CZTS and GeSe, to form heterojunctions in our previous work^25,43^. Accompanied by the modification of the CBD-CdS layer, the photocurrent density and onset potential of the Pt-CdS/Cu_3_BiS_3_ photocathodes were substantially improved to ~2 mA/cm^2^ (at 0 V_RHE_) and ~0.75 V_RHE_, respectively. The enhancement of the PEC performance was attributed to the heterojunction that was formed at the interface of CdS/Cu_3_BiS_3_, which can efficiently promote the separation of charge carriers and reduce surface recombination ^25,43^.

Nevertheless, the photocurrent density of the Pt-CdS/Cu_3_BiS_3_ photocathode was less than half of the initial value after the 3 h stability test, as shown in Fig. 3d, due to the oxidative self-photocorrosion of the CdS layer^25^. Consequently, a protective TiO_2_ layer deposited by atomic layer deposition (ALD) was modified onto the surface of the CdS layer to achieve relatively stable photocathodes in this work, which is a conventional strategy to enhance the stability of previously reported photocathodes such as CZTS^44^, Cu_2_BaSn(S, Se)_4_^45^, Cu(In,Ga)Se_2_^13^, GeSe^43^, Sb_2_Se_3_^41^ and Cu_2_O^31^. To our surprise, as shown in Fig. 3a and d, not only was the stability of the photocathodes significantly improved to over 10 h but also the photocurrent density dramatically increased from ~2 mA/cm^2^ to ~7 mA/cm^2^ (at 0 V_RHE_), and the onset potential increased to above ~0.9 V_RHE_ with the modification of the TiO_2_ layer. Meanwhile, there were no obvious corrosive metamorphisms at the surface of the finished photocathodes by comparing the cross-sectional SEM images before and after the 10 h stability test (Fig. S8), further confirming the great protective effect of the pinhole-free ALD-TiO_2_ layer. It should be emphasized that such a relatively high onset potential of ~0.9 V_RHE_ provides the conditions for the realization of efficient unbiased water splitting cells in contact with appropriate photoanodes such as BiVO_4_ and Fe_2_O_3_^29,30^. Moreover, the finished photocathode achieved the highest ABPE of 1.7% at 0.4 V_RHE_, which was more than four times that of the Pt-CdS/Cu_3_BiS_3_ photocathode, as shown in Fig. 3b.

The protective effect of the TiO_2_ layer can be referenced to previous related works^13,31,41,43,45^, and the underlying mechanism of the increased PEC performance of the inserted TiO_2_ layer was further understood through a series of PEC characterizations and band schema. Figure 3c shows the spectra of the incident photocurrent conversion efficiency (IPCE) undertaken at 0 V_RHE_ and the corresponding integrated photocurrent density of the Pt-CdS/Cu_3_BiS_3_ and Pt-TiO_2_/CdS/Cu_3_BiS_3_ photocathodes. Accompanied by the importation of the TiO_2_ layer, the increase in the IPCE in the low wavelength range was more obvious than that in the relatively long wavelength range (the IPCE value increased from 15 to 44% at 450 nm), indicating that the inserted TiO_2_ layer greatly enhanced the light harvesting and conversion efficiency in the low wavelength range of the finished photocathode. It was observed that the efficiency dropped suddenly in the wavelength range of 490–540 nm with a long absorption tail after 590 nm for the finished photocathodes, suggesting that the interfacial recombination of photoexcited carriers in the Pt-TiO_2_/CdS/Cu_3_BiS_3_ photocathode was still obvious. Whether the TiO_2_ layer was introduced or not, the integrated photocurrent density practically matched the photocurrent density measured by LSV, demonstrating the uniformity of LSV measurements and IPCE results. In addition, the reflection characterizations (Fig. S9) of our Cu_3_BiS_3_-based photocathodes with and without TiO_2_ layer also demonstrated that the TiO_2_ layer can be used as an antireflection layer to enhance the light absorption for the Cu_3_BiS_3_-based photocathodes.

Furthermore, intensity-modulated photovoltage spectroscopy (Fig. S10) and intensity-modulated photocurrent spectroscopy (Fig. S11) of the three electrodes (Pt-Cu_3_BiS_3_, Pt-CdS/Cu_3_BiS_3_, and Pt-TiO_2_/CdS/Cu_3_BiS_3_) were performed to understand the surface/interfacial recombination or transfer of photogenerated carriers in the electrodes. Through the calculation (the detailed computational procedures are given in the Supporting Information), the Pt-TiO_2_/CdS/Cu_3_BiS_3_ photocathode exhibited a higher carrier lifetime (*τ*_*n*_) and a lower charge transfer time (*τ*_*d*_) than those of the Pt-Cu_3_BiS_3_ and Pt-CdS/Cu_3_BiS_3_ photocathodes, which indicated that the transfer efficiency of the photoexcited carriers was enhanced and the interfacial recombination rate was decreased with the importation of the TiO_2_/CdS overlayer ^46^.

The band diagram of the TiO_2_/CdS/Cu_3_BiS_3_ electrode was further investigated, as shown in Fig. S12. There was a conspicuous cliff-like conduction band offset (0.7 eV) at the interface of CdS/Cu_3_BiS_3_, which would result in a recombination center at the interface and be unbeneficial for the transfer of the photogenerated electrons^33^. This is also one of the reasons why the previously reported Cu_3_BiS_3_-based photovoltaic devices presented a poor conversion efficiency buffered with a CdS layer^35,37^. Accompanied by the inserted ALD-TiO_2_ layer, the photogenerated electrons and holes can be separated more effectively due to the relatively low valence band position of TiO_2_. Moreover, the photogenerated electrons can also be selectively transferred through the TiO_2_ layer directly into the electrolyte and participate in the hydrogen evolution reaction^24^. Based on the above discussion, these factors all led to a higher PEC performance for the Pt-TiO_2_/CdS/Cu_3_BiS_3_ photocathode than the photocathode modified with a single CdS layer.

Figure 3e shows the H_2_ and O_2_ production amounts of the finished photocathode biased at 0 V_RHE_ with various illumination times. H_2_ gas was produced with a constant rate of 141 µmol cm^−2^ h^−1^, indicating the highly stable overall solar water splitting properties of the Pt-TiO_2_/CdS/Cu_3_BiS_3_ photocathode. Notably, the H_2_ and O_2_ production amounts of the finished photocathode biased at more positive potentials (such as 0.3 V_RHE_ and 0.6 V_RHE_) were also determined, and as shown in Fig. S13, H_2_ gas can be produced steadily with constant rates of 95.7 µmol cm^−2^ h^−1^ and 35.2 µmol cm^−2^ h^−1^, respectively, when the finished photocathode was biased at 0.3 V_RHE_ and 0.6 V_RHE_ under simulated solar irradiation, which further identified the authenticity of such a high onset potential (~0.9 V_RHE_) of the Pt-TiO_2_/CdS/Cu_3_BiS_3_ photocathode. Meanwhile, according to our calculation, the faradaic efficiency of this Pt-TiO_2_/CdS/Cu_3_BiS_3_ photocathode biased at any potential (0 V_RHE_, 0.3 V_RHE_ and 0.6 V_RHE_) were all close to 90% (Fig. S14). Figure 3f is a photograph of the Pt-TiO_2_/CdS/Cu_3_BiS_3_ photocathode under working conditions.

The thickness of the CdS buffer was found to significantly influence the PEC properties of the Cu_3_BiS_3_-based photocathode, and the statistical results are shown in Fig. S15. It was found that the optimum thickness of the CdS buffer was ~80 nm (i.e., 15 min CBD). A thinner CdS buffer (i.e., <80 nm) may not efficiently passivate the surface of Cu_3_BiS_3,_ while a thicker CdS buffer (higher than 80 nm) would increase the resistance and absorb solar light without passing through the Cu_3_BiS_3_ photoabsorber (Fig. S16). In addition, a CdS layer that is too thick may increase the photoexcited carrier recombination efficiency, as shown in the transit photocurrent spectra (Fig. S17). On the basis of these studies, the optimum deposition time of the CBD-CdS layer was determined to be 15 min in this work, and the thickness of the CBD-CdS layer deposited for 15 min can reach ~80 nm (Fig. S7)

### Scalable Cu_3_BiS_3_-BiVO_4_ tandem cell for unbiased solar water splitting

**Fig. 4:**
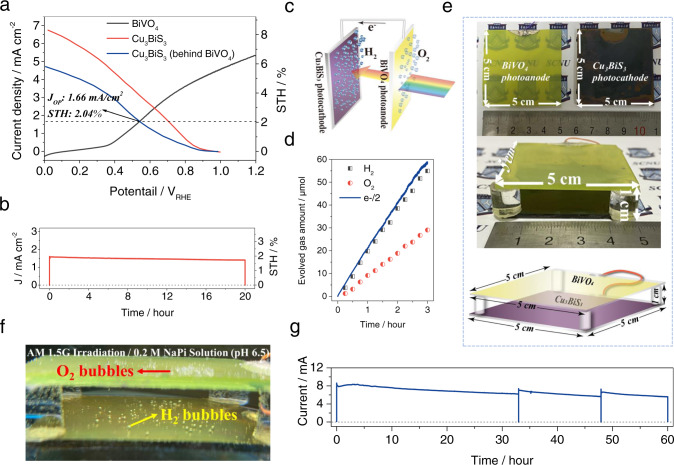
PEC performance of the scalable Cu_3_BiS_3_-BiVO_4_ tandem device. (**a**) J–V curves of the Cu_3_BiS_3_ photocathode, BiVO_4_ photoanode and Cu_3_BiS_3_ photocathode behind the BiVO_4_ photoanode. (Jop: the operation point photocurrent density, STH: solar to hydrogen efficiency). (**b**) Photocurrent density-time curve, (**c**) working diagram and (**d**) hydrogen and oxygen evolution amount-time curves of the Cu_3_BiS_3_-BiVO_4_ tandem device. (**e**) Photographs of the 5 × 5 cm^2^ Cu_3_BiS_3_ photocathode, 5 × 5 cm^2^ BiVO_4_ photoanode and large area (5 × 5 cm^2^) Cu_3_BiS_3_-BiVO_4_ tandem device and diagram of the large area (5 × 5 cm^2^) Cu_3_BiS_3_-BiVO_4_ tandem device. (**f**) Working photograph of the large area (5 × 5 cm^2^) Cu_3_BiS_3_-BiVO_4_ tandem device under solar simulated AM 1.5 G irradiation. (**g**) Photocurrent density-time curve of the large area (5 × 5 cm^2^) Cu_3_BiS_3_-BiVO_4_ tandem device. All measurements were carried out in a 0.2 mol/dm^3^ Na_2_HPO_4_/NaH_2_PO_4_ solution (pH = 6.5) under solar simulated AM 1.5 G irradiation. Source data are provided as a Source Data file.

It should be noted here that the Cu_3_BiS_3_-based photocathode presented a very promising solar water splitting photovoltage >0.9 V_RHE_, and such a high onset potential is very important and suitable to assemble efficient tandem of photocathode-photoanode cells for standalone devices. In this work, we prepared a tandem cell of Cu_3_BiS_3_-BiVO_4_. Figure 4a shows the current-potential curves obtained from a Cu_3_BiS_3_-based photocathode and a BiVO_4_ photoanode; thus, a two-electrode tandem PEC cell was assembled by placing a BiVO_4_/FTO photoanode (0.73 cm^2^) in front of a Pt-TiO_2_/CdS/Cu_3_BiS_3_ photocathode (0.45 cm^2^) followed by exposure to simulated AM 1.5 G solar radiation, as shown in Fig. S18. The details of the synthesis of the efficient BiVO_4_ photoanode were illustrated in our previous paper^29^. There was an obvious duplication between the *J–V* curves of the Cu_3_BiS_3_ photocathode and BiVO_4_ photoanode under a photovoltage from 0.35 V_RHE_ to 0.9 V_RHE_, and the crossing point and operation point were observed at 1.66 mA/cm^2^ (*J*_*op*_) and 0.54 V_RHE_, respectively. We showed the video of the Cu_3_BiS_3_-BiVO_4_ tandem cell under working with simulated sunlight irradiation (Movie S1) in Supporting Information, many H_2_/O_2_ bubbles were evolved from the tandem cell for the overall solar water splitting reaction. Meanwhile, the IPCE spectra (Fig. S19) measured under the operating bias (0.54 V_RHE_) for both electrodes show complimentary spectral responses. The energy band diagrams of the Cu_3_BiS_3_-BiVO_4_ tandem cell (Fig. S20) show the photogenerated carrier transfer path and solar hydrogen evolution reaction process. Based on the STH efficiency calculation equation:

1 $${\eta }_{STH}=({J}_{op}\times 1.23)/P$$

where *P* is the power of the illuminating light, the STH of this Cu_3_BiS_3_-BiVO_4_ tandem cell achieved a remarkable efficiency of 2.04%. This 2.04% STH efficiency is the first reported and recorded unbiased STH efficiency for Cu_3_BiS_3_-BiVO_4_ tandem cells, which is very close to the record STH efficiency from Cu_2_O-BiVO_4_ tandem cells reported by the Gratzel group (Table 1) ^31^.

**Table 1:** PEC performance of previously reported typical photocathodes and their tandem cell modules.

Typical photocathodes	Electrolyte pH	J /mA cm^-2^; ABPE; Stability (retention)	Onset potential /V_RHE_	Tandem cell modules	Tandem cell STH	Tandem cell stability (retention)	Reference; Year [Ref.]
Cu_3_BiS_3_/CdS/TiO_2_-Pt	6.5	−7.0 (0 V_RHE_); 1.7%; 10 hours (90%)	0.9	Cu_3_BiS_3_-BiVO_4_	2.04%	20 hours (90%)	this work; 2021
Cu_2_O-RuO_x_	6.0	−2.6 (0 V_RHE_); NR; NR	0.55	Cu_2_O-BiVO_4_	1.23%;	1.7 minutes (40%)	Bornoz et al; 2014^47^
CuIn_0.5_Ga_0.5_Se_2_/CdS-Pt	6.8	−28.0 (0 V_RHE_); 12.5%; NR	0.7	CIGS-BiVO_4_	3.7%	10 minutes (81%)	Kobayashi et al; 2018^12^
Zn:InP NW/TiO_2_-Pt	1.0	−18.0 (0 V_RHE_); 4%; 10 hours (60%)	0.6	InP-BiVO_4_	0.5%	1 minute (85%)	Kornienko et al; 2016^48^
CIGS/CdS/Al_2_O_3_/TiO_2_-P-S-Pt	6.8	−26.0 (0 V_RHE_); 6.6%; 8 hours (95.5%)	0.6	CIGS-BiVO_4_	1%	0.5 hour (85%)	Chen et al; 2018^13^
Si(n+-p)-Mo-Ni	1.0	−35.5 (0 V_RHE_); NR; NR	0.495	Si(n^+^-p)-BiVO_4_	2.1%;	1 hour (100%)	Vijselaar et al; 2018^10^
CuBi_2_O_4_	6.8	−3.7 (0.4 V_RHE_); NR; NR	1.0	CuBi_2_O_4_-BiVO_4_	0.86%	1 hour (86%)	Song et al; 2020^49^
(Ag,Cu)GaSe_2_/CuGa_3_Se_5_/CdS-Pt	7.0	−7.8 (0 V_RHE_); 1.4%; NR	0.8	CGS/ACGSe-BiVO_4_	0.67%	2 hours (99%)	Kim et al; 2016^22^
Perovskite/IO-TiO_2_-H_2_ase	6.0	−5.0 (0 V_RHE_); NR; 12 hours (67%)	0.8	Perovskite-BiVO_4_	1.1%	10 hours (22%)	Moore et al; 2020^50^
Perovskite/Field’s metal-Pt	8.5	−12.1 (0 V_RHE_); NR; 7 hours (75%)	0.8	Perovskite-BiVO_4_	0.35%	18 hours (71%)	Andrei et al; 2018^51^
(ZnSe)_0.85_(CIGS)_0.15_/CdS/In_2_S_3_-RuO_2_	9.5	NR;NR;NR	NR	(ZnSe)_0.85_(CIGS)_0.15_-BiVO_4_	1%	50 hours (67%)	Kaneko et al; 2018^52^
Cu_2_O/Ga_2_O_3_/TiO_2_-RuO_x_	5.0	−10.0 (0 V_RHE_); NR; 120 hours (80%)	1.0	Cu_2_O-BiVO_4_	3%	12 hours (90%)	Pan et al; 2018^31^
Sb_2_Se_3_/CdS/TiO_2_-Pt	1.0	−20.0 (0 V_RHE_); 3.4%; NR	0.5	Sb_2_Se_3_-BiVO_4_	1.5%	10 hours (90%)	Yang et al; 2020^18^
CZTS/CdS/HfO_2_-Pt	6.5	−12.0 (0 V_RHE_); 2.7%; 10 hours (90%)	0.65	CZTS-BiVO_4_	1.05%	10 hours (87%)	Jiang et al; 2018^25^
CZTS/HfO_2_/CdS/HfO_2_-Pt	6.5	−18.0 (0 V_RHE_); 5.6%; 24 hours (97%)	0.72	CZTS-BiVO_4_	3.17%	20 hours (85%)	Jiang et al; 2021^53^

PEC, photoelectrochemical; ABPE, applied bias photon-to-current efficiency; STH, solar to hydrogen efficiency; NR, not reported.

Stability is another important issue to evaluate the real application potential, and we characterized the long-term photocurrent stability of this Cu_3_BiS_3_-BiVO_4_ tandem cell over 20 h of irradiation (AM 1.5 G). The data shown in Fig. 4b demonstrate that the tandem cell possessed a very high long-term stability, and the photocurrent after 20 h of irradiation maintained ~90% of its initial value. The working diagram of this tandem cell is shown in Fig. 4c. In addition, Fig. 4d shows that the Cu_3_BiS_3_-BiVO_4_ tandem cell possessed stable H_2_ gas evolution with a constant ratio of 18 µmol/h.

The appreciable efficiency and high stability of the Cu_3_BiS_3_ photocathode and the Cu_3_BiS_3_-BiVO_4_ tandem cell are very suitable for practical utilizations. However, the device size is another key point. Fortunately, large Cu_3_BiS_3_ and BiVO_4_ photoelectrodes are not difficult to obtain. We prepared 5 × 5 cm^2^ Cu_3_BiS_3_ and BiVO_4_ and assembled them into a tandem device in this work, and their photos and the model diagram are shown in Fig. 4e. The 5 × 5 cm^2^ size Cu_3_BiS_3_ photocathode not only presented an appreciable photocurrent of about 90 mA under 0 V_RHE_ (active area: 21 cm^2^) but also possessed an excellent working stability (Fig. S21). Significant number of H_2_ bubbles were observed to be continuously produced from the surface of Cu_3_BiS_3_ photocathode under simulated sunlight irradiation (Movie S2, Fig. S22). It should be noted here that 5 × 5 cm^2^ is the largest size we can currently prepare due to the limit of our apparatus, and much larger sizes, such as 10 × 10 cm^2^ or 20 × 20 cm^2^, should be able to be prepared when we update our apparatus. Figure 4f and Movie S3 show a photograph and a video of this 5 × 5 cm^2^ Cu_3_BiS_3_-BiVO_4_ tandem device under simulated AM 1.5 G solar light illumination, respectively. Many H_2_ and O_2_ bubbles can be observed on the surface of the Cu_3_BiS_3_ photocathode and BiVO_4_ photocathode, respectively. As shown in Fig. 4g, the photocurrent of this 5 × 5 cm^2^ Cu_3_BiS_3_-BiVO_4_ tandem device did not obviously decrease even over the 60 h stability test, indicating that the long-term operation of the large Cu_3_BiS_3_-BiVO_4_ tandem device is promising.

**Fig. 5:**
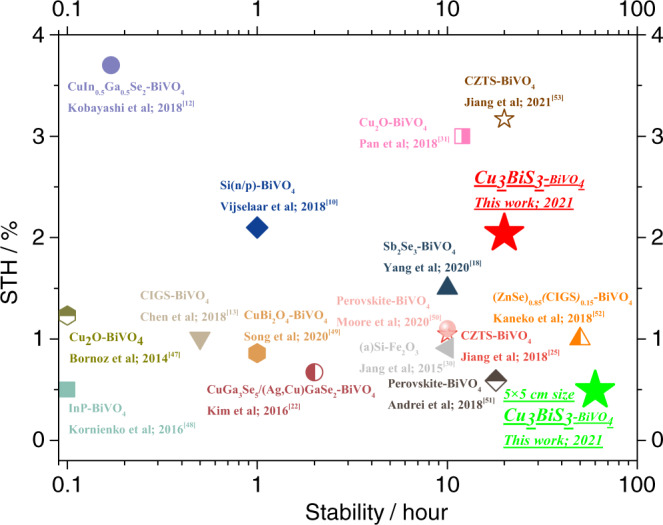
Benchmarks for previously reported photocathode-photoanode tandem cells. Efficiency and stability benchmarks of the previously reported photocathode-photoanode tandem cells for standalone water splitting. The statistical PEC performance parameters are shown in Table 1.

Although the photocurrent of the Cu_3_BiS_3_-based photocathodes in this work still has much room for improvement, the photovoltage and stability of the Cu_3_BiS_3_ photocathode presented here are very close to the record of the Cu_2_O photocathode^31^ but already higher than those of various typical photocathodes, such as CZTS^25^, CIGS^12^, Sb_2_Se_3_^18^, Si^10^ and so on (Table 1). We can expect large potential for the improvement in the PEC properties of Cu_3_BiS_3_-based photocathodes. Compared with Cu_2_O, we found that the Cu_3_BiS_3_ film is relatively stable in neutral buffer solutions and that the stable Cu_3_BiS_3_ film is much easier to obtain than Cu_2_O^31^. We believe that the Cu_3_BiS_3_-based photocathode will attract much attention and be significantly improved in the near future. The over 2% STH efficiency of the Cu_3_BiS_3_-BiVO_4_ tandem devices and over 20 h of stability presented indicate great potential for solar water splitting. In comparison with the previously reported typical tandem cell (Fig. 5), it was found that the STH efficiency of our Cu_3_BiS_3_-BiVO_4_ tandem cell was competitive among them. Nevertheless, it should be noted that the research of the Cu_3_BiS_3_ photoelectrode is almost at the starting point now, and the presented appreciable STH efficiency (2.04%) and stability (20 h and 60 h) indicate great potential for Cu_3_BiS_3_ photoelectrodes. We believe that the PEC efficiency/stability of the Cu_3_BiS_3_ photoelectrode and Cu_3_BiS_3_-BiVO_4_ tandem cell will be significantly improved and even higher than those of other famous CIGS-, Cu_2_O- and CZTS-based photoelectrodes in the near future. This work provides a promising starting point for the development of Cu_3_BiS_3_-based photoelectrodes, and we believe that one milestone after another will be achieved in the future.

## Discussion

In this work, the Cu_3_BiS_3_-based photocathode exhibited excitation at a set potential over 0.9 V_RHE_ and an excellent PEC current density of ~7 mA/cm^2^ under 0 V_RHE_ with 10 h long-term high stability in neutral water solutions. Further fabricated Cu_3_BiS_3_-BiVO_4_ photocathode-to-photoanode tandem cells presented a good unbiased STH conversion efficiency as high as 2.04%. However, such tandem devices also presented superior high stability over 20 h. Finally, a 5 × 5 cm^2^ large Cu_3_BiS_3_-BiVO_4_ tandem device module was fabricated for scalable overall solar water splitting device applications, and the stable photocurrent of this larger module over 60 h of operation under sustained solar light irradiation was detected. The appreciable PEC properties, such as the high onset potential, good photocurrent, over 60 h of high stability, ideal STH efficiency (2.04%) and scalable device module (5 × 5 cm^2^) for the Cu_3_BiS_3_-based photocathode that were presented in this work, all indicate its remarkable application value and great potential for solar water splitting.

## Methods

### Preparation of the Cu_3_BiS_3_ films

The Cu_3_BiS_3_ films (with a Cu/3:Bi = 1 mole ratio) were prepared by spray pyrolysis method. The sprayed precursor solution was mixed by 15 mL CuCl-Tu (excess) DMSO solution and 15 mL BiCl_3_- Tu (excess) DMSO solution. Specifically, the Cu-Tu stock solution was formed by 2.97 g CuCl (2 M) and excess thiourea (Tu) both dissolved in 15 mL DMSO, and the Bi-Tu stock solution was formed by 3.15 g BiCl_3_ (0.666 M) and excess thiourea (Tu) both dissolved in 15 mL DMSO. Then, the two stock solutions were both separately stirred until clear and transparent before their mix. Furthermore, the mixing CuCl (1 M), BiCl_3_ (0.333 M) and excess Tu precursor solution was also stirred for 5 h, ultimately obtaining a yellow clear sprayed solution for Cu_3_BiS_3_ films preparation. The precursor solution was subsequently sprayed onto a cleaned Mo-coated soda-lime glass substrate preheated to 380 °C for about 3 min.

### Surface mdification with TiO_2_/CdS overlayer

First, a CdS layer was deposited under the Cu_3_BiS_3_ layer by the chemical bath deposition method (CBD). The prepared Cu_3_BiS_3_ layer was dipped into an aqueous solution containing 12.5 mM CdSO_4_, 0.22 mM SC(NH_2_)_2_, and 11 M NH_4_OH at 60 °C for 15 min.

Furthermore, a TiO_2_ layer was deposited under the CdS/Cu_3_BiS_3_ double layer by ALD method. The TiO_2_ layer was grown by using titanium tetrakis (dimethylamide) as titanium source and H_2_O as oxygen source. Based on our empirical value, the growth rate was estimated to be about 0.054 nm per cycle and the TiO_2_ film was grown for 926 cycles at 120 °C, and the corresponding thicknesses of the TiO_2_ layer was 50 nm, ultimately obtaining a TiO_2_/CdS/Cu_3_BiS_3_ electrode.

### Deposition of Pt particles

The Pt particles deposition was performed by using a three-electrode system consisting of TiO_2_/CdS/Cu_3_BiS_3_ as a working electrode, a Pt wire as a counter electrode, and Ag/AgCl as a reference electrode. These electrodes were put in 0.1 M Na_2_SO_4_ solution containing 1 mM H_2_PtCl_6_, and the deposition was performed with a constant potential of −0.1 V_Ag/AgCl_ by using CHI660E electrochemical measurement unit. During the deposition process, the working electrode was illuminated by simulated AM 1.5 G solar irradiation.

### Preparation of the BiVO_4_ photoanodes

The BiVO_4_ films were also prepared by spray pyrolysis method. The sprayed solution was prepared by dissolving Bi(NO_3_)_3_·5H_2_O in acetic acid and VO(AcAc)_2_ in absolute ethanol. The Bi stock solution was then added into the V stock solution, and the mixture was diluted to 4 mM with excess ethanol. The mixed precursor solution was immediately sprayed onto a cleaned FTO-coated glass substrate preheated to 450 °C, and the spray nozzle was placed 25 cm above the heating plate. The sprayed solution was driven by an over pressure of 0.5 bar of N_2_ gas.

### Photoelectrochemical measurements

An online gas chromatography system (Shimadazu GC-2014 gas analyzer equipped with a MS-5A column and a thermal conductivity detector) was used to detect H_2_ and O_2_ during the PEC water splitting. PEC H_2_ generation from photocathodes was examined in a pH 6.5 phosphate buffer solution (0.2 M Na_2_HPO_4_/NaH_2_PO_4_) by using the above-mentioned solar simulator as a light source. The PEC cell was covered by a water jacket to maintain the temperature at 293 K. The two-electrode setup composed of the Pt-TiO_2_/CdS/Cu_3_BiS_3_ photocathode and a BiVO_4_ photoanode in series connection was also employed to examine water splitting under the bias-free condition. Furthermore, the phosphate buffer solution was updated per 20 h stability test to keep the concentration (0.2 M) and pH (6.5) unchanged. The intensity of simulated sunlight was also calibrated per 20 h stability test. Potentials referred to the Ag/AgCl electrode were converted to reversible hydrogen electrode using the Nernst equation:

2 $${V}_{RHE}={V}_{Ag/AgCl}+0.059\times pH+0.199$$

ABPE was determined from the current density−potential response of the photocathodes by using the following equation:

3 $${\rm{ABPE}}\, ( \% )=J\times V\times 100/P$$

Where *J* is the photocurrent density (mA/cm^2^), *V* is the applied potential (V_RHE_), and *P* is the intensity of simulated sunlight (100 mW/cm^2^).

### Structural characterization

Crystalline structures of the films were determined by XRD, Raman spectroscopy and TEM using a Rigaku Mini Flex X-ray diffractometer, a Jasco NRC 3100 laser Raman spectrophotometer and JEOL JEM-2100HR microscope respectively. Surface and cross section morphology were observed by scanning electron microscope (SEM) using Hitachi S-4800 micros.

## Supplementary information

Supplementary Information pdf file

Peer review

Supplementary Movie S1

Supplementary Movie S2

Supplementary Movie S3

Description of additional supplementary files

## Data Availability

The data that support the plots and other findings of within this paper are available from the corresponding authors on reasonable request. Source data are provided with this paper.

## Source data

source data
