# Supplementary material for: Wittichenite semiconductor of Cu3BiS3 films for efficient hydrogen evolution from solar driven photoelectrochemical water splitting
Source: Nat Commun. 2021 Jun 18;12:3795. doi: 10.1038/s41467-021-24060-5 (PMC8213846; doi:10.1038/s41467-021-24060-5)
Supplement: Supplementary file 1 — Supplementary Information pdf file [file 41467_2021_24060_MOESM1_ESM.pdf]

Supplementary Information

Wittichenite semiconductor of Cu_3BiS_3 films for efficient hydrogen evolution from solar driven photoelectrochemical water splitting

Dingwang Huang[a], Lintao Li[a], Kang Wang[a], Yan Li[a], Kuang Feng[a] and Feng Jiang[a,b,c]*

a. Institute of Semiconductor Science and Technology, South China Normal University, Guangzhou 510631, P. R. China

b. SCNU Qingyuan Institute of Science and Technology Innovation Co., Ltd., Qingyuan 511517, China

c. Guangdong Provincial Engineering Technology Research Center for Low Carbon and Advanced Energy Materials, South China Normal University, Guangzhou, 510631, China

*email address: fengjiangsolar@126.com

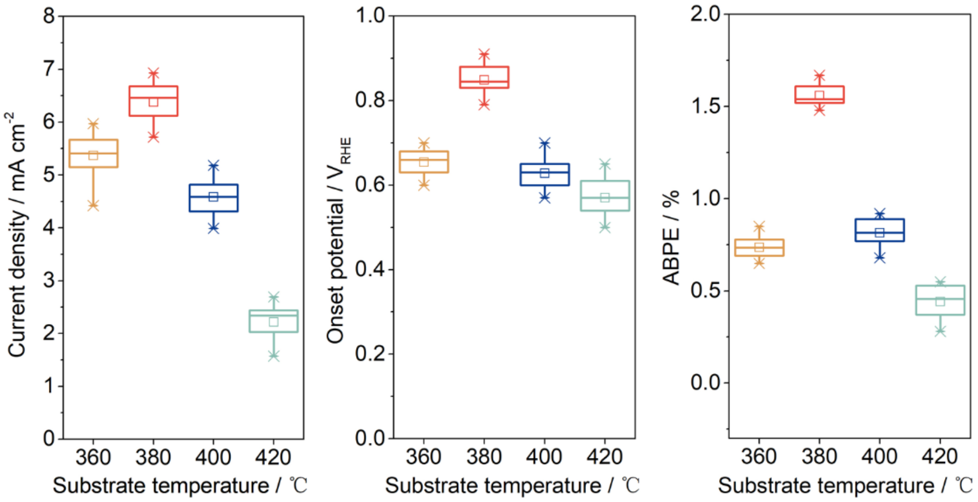

Fig. S1 Statistical box data of PEC performance (photocurrent density, onset potential and ABPE) for the Cu_3BiS_3-based photocathodes sprayed at various substrate temperatures (360°C, 380°C, 400°C and 420°C). The box plot denotes median (centre line), mean value (dots), 25th (bottom edge of the box), 75th (top edge of the box), 95th (upper whisker) and 5th (lower whisker) percentiles. The sample size in each column is 10 devices. Source data are provided as a Source Data file.

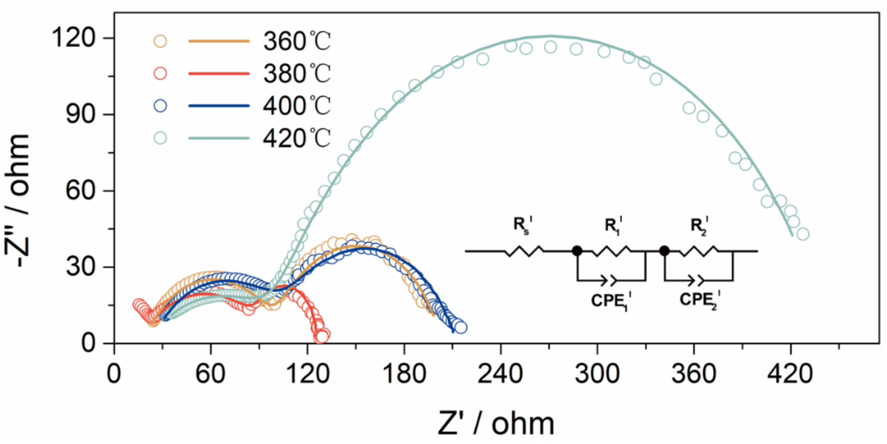

Fig. S2 Nyquist plots (biased at 0.3 V_{RHE} under AM 1.5G light irradiation with the frequency range of 100 kHz to 0.1 Hz) of the Cu_3BiS_3-based photocathodes sprayed at various substrate temperatures (360°C, 380°C, 400°C and 420°C). In the Nyquist plots, scatter points represent the original experimental data, whereas solid lines represent fitted curves based on the equivalent circuit shown in the inset. Source data are provided as a Source Data file.

Table S1 Fitted EIS resistance parameters of the Cu_3BiS_3 photocathodes sprayed at virous substrate temperatures (360℃, 380℃, 400℃ and 420℃) based on the circuits shown in the inset of Fig. S2.

Samples at various substrate temperature	R_S / Ω cm^2	R_1 / Ω cm^2	R_2 / Ω cm^2
360℃	18.08	83.41	101.5
380℃	14.27	82.17	30.44
400℃	22.86	83.95	105.77
420℃	20.46	84.4	339.3

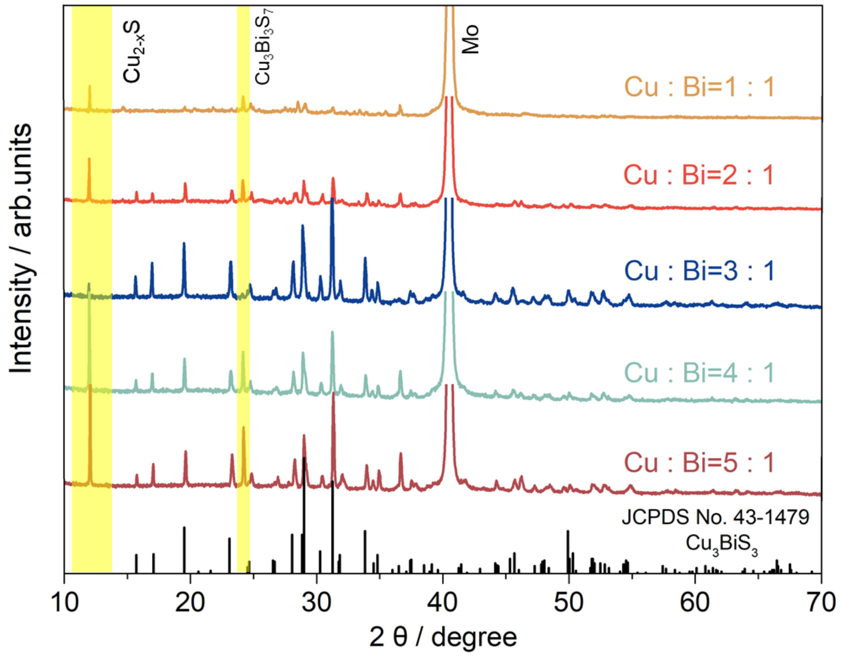

Fig. S3 XRD patterns of the Cu_3BiS_3 films sprayed from the precursor solution with various Cu : Bi molar ratios (1 : 1, 2 : 1, 3 : 1, 4 : 1 and 5 : 1). Source data are provided as a Source Data file.

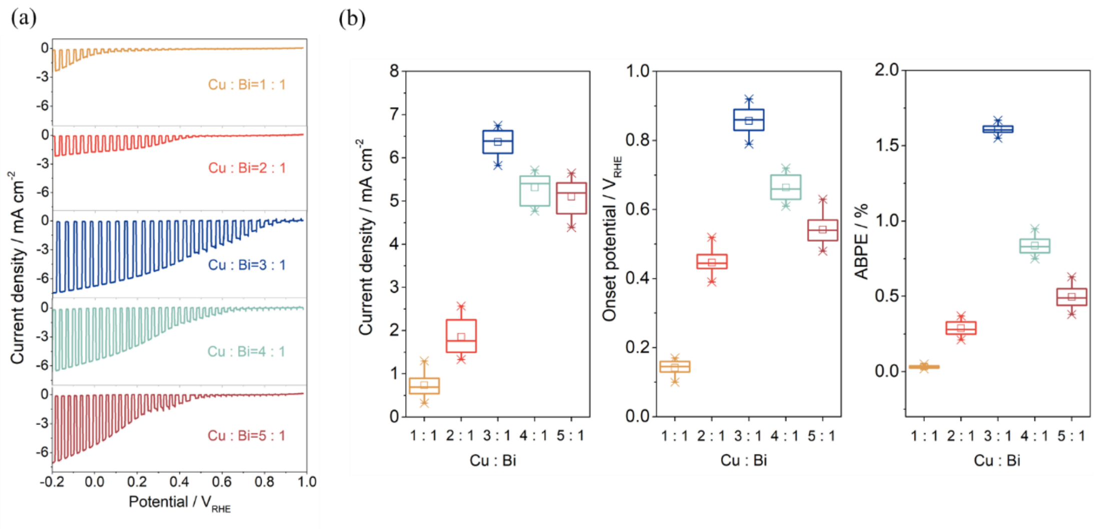

Fig. S4 **a** Chopped photocurrent density-potential curves of the Cu_3BiS_3-based photocathodes sprayed with various Cu : Bi molar ratios (1 : 1, 2 : 1, 3 : 1, 4 : 1 and 5 : 1) and **b** corresponding statistical box data of the PEC performance (photocurrent density, onset potential and ABPE). The box plot denotes median (centre line), mean value (dots), 25th (bottom edge of the box), 75th (top edge of the box), 95th (upper whisker) and 5th (lower whisker) percentiles. The samples size in each column is 10 devices. Source data are provided as a Source Data file.

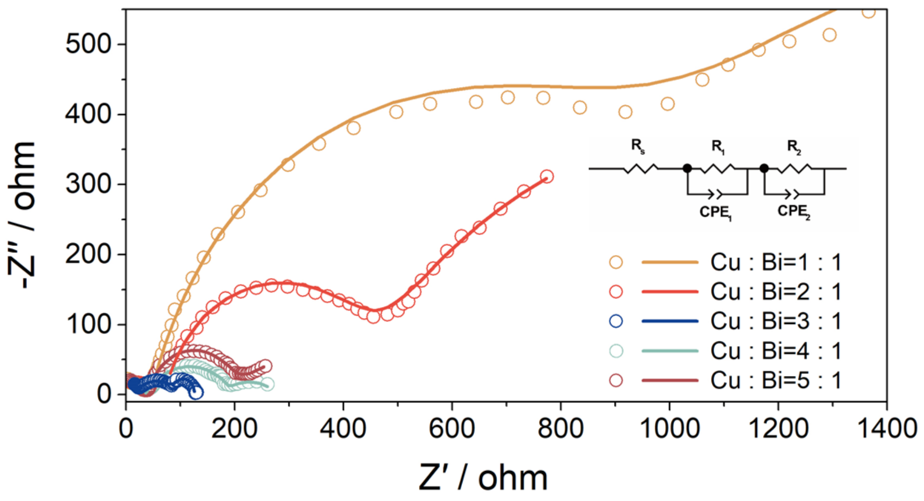

Fig. S5 Nyquist plots (biased at 0.3 V_{RHE} under AM 1.5G light irradiation with the frequency range of 100 kHz to 0.1 Hz) of the Cu_3BiS_3-based photocathodes sprayed with various Cu : Bi molar ratios (1 : 1, 2 : 1, 3 : 1, 4 : 1 and 5 : 1). In the Nyquist plots, scatter points represent the original experimental data, whereas solid lines represent fitted curves based on the equivalent circuit shown in the inset. Source data are provided as a Source Data file.

Table S2 Fitted EIS resistance parameters of the Cu_3BiS_3-based photocathodes sprayed with virous Cu : Bi molar ratios (1 : 1, 2 : 1, 3 : 1, 4 : 1 and 5 : 1) based on the circuits shown in the inset of Fig. S5

Samples with various Cu : Bi mole ratios	R_S ´/ Ω cm^2	R_1´ / Ω cm^2	R_2 ´/ Ω cm^2
Cu : Bi=1 : 1	44.93	823.8	2183
Cu : Bi=2 : 1	70.12	368.5	1304
Cu : Bi=3 : 1	14.27	82.17	30.44
Cu : Bi=4 : 1	33.85	166.5	74.42
Cu : Bi=5 : 1	34.99	142.3	1610

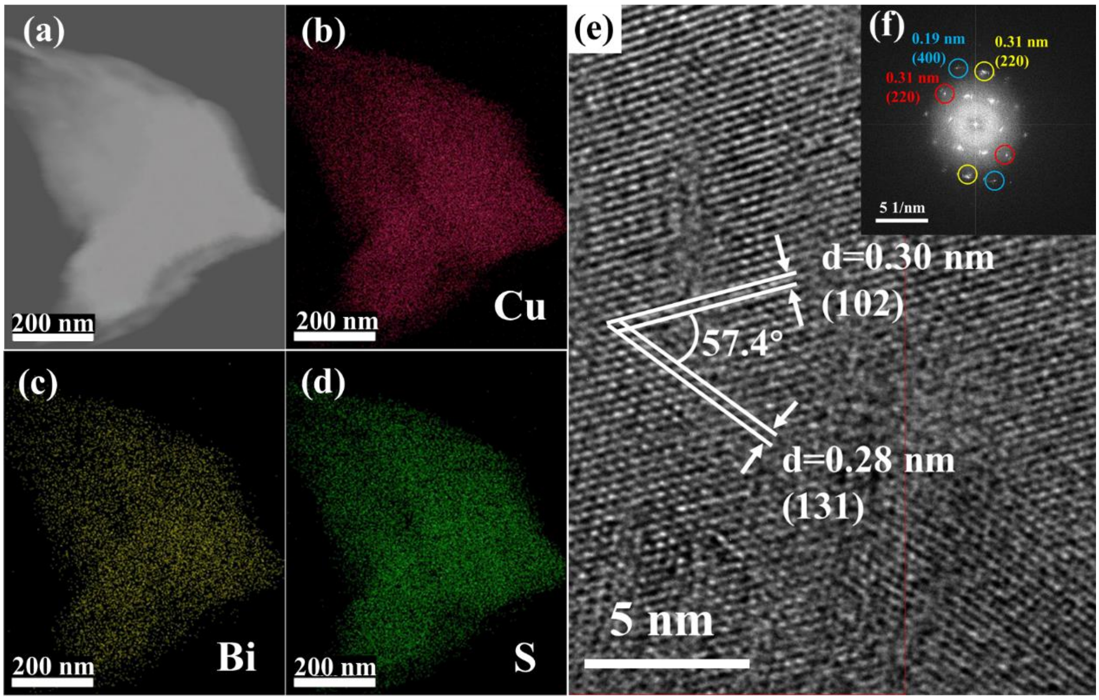

Fig. S6 **a** TEM image of the prepared Cu_3BiS_3 grain, and its corresponding STEM-EDX elemental mappings of element **b** Cu, **c** Bi, **d** S, **e** HRTEM image of Cu_3BiS_3 crystal, and **f** its SAED pattern.

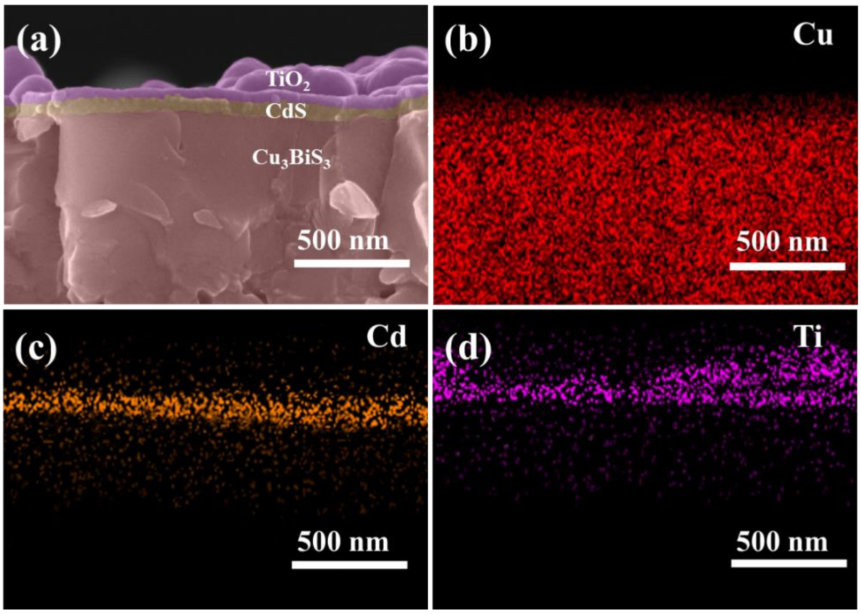

Fig. S7 a Cross-sectional SEM images of the Pt-TiO_2/CdS/Cu_3BiS_3 electrode; EDS mapping for the elements of **b** Cu, **c** Cd and **d** Ti in the whole area of **a**.

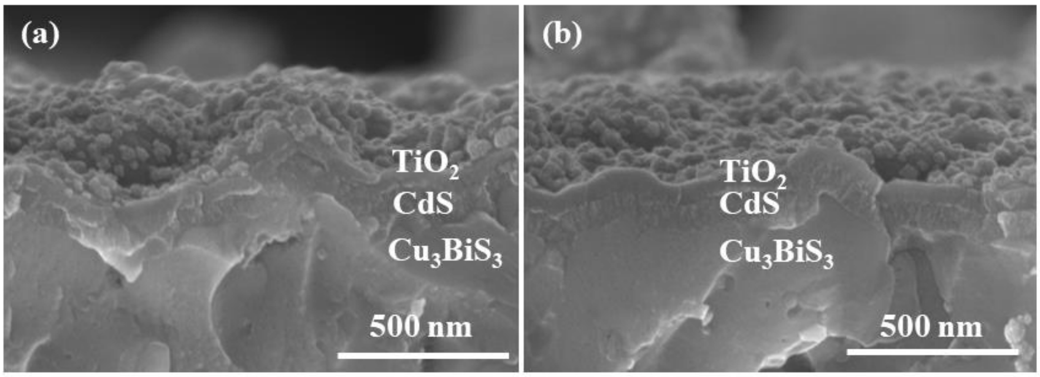

Fig. S8 a Cross-sectional SEM images of the Pt-TiO_2/CdS/Cu_3BiS_3 electrode before stability test and **b** after 10 hours stability test.

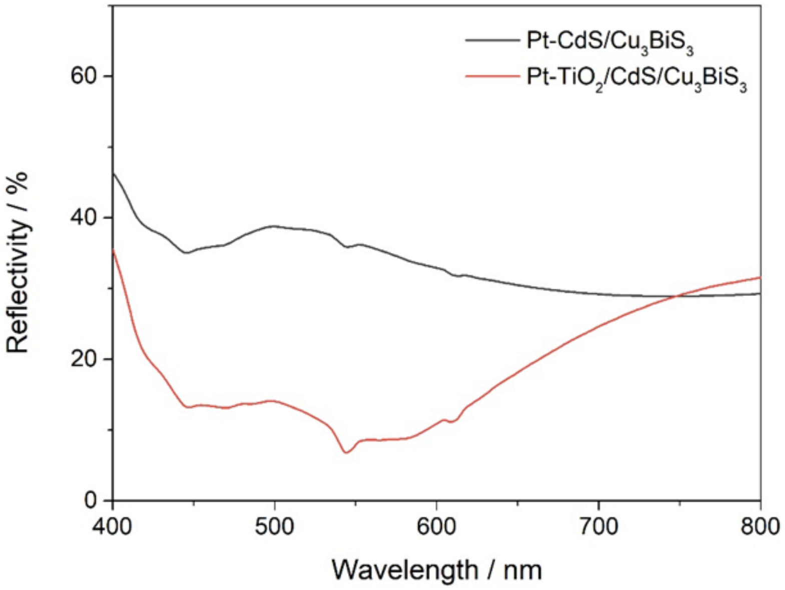

Fig. S9 Reflectivity curves of the Cu_3BiS_3-based photocathodes with and without TiO_2 layer. Source data are provided as a Source Data file.

As shown in Fig. S9, it is obvious that the reflectively of the Pt-CdS/Cu_3BiS_3 photocathodes is relatively high than that modified with TiO_2 layer, indicating that the TiO_2 layer can be used not only as a protective layer but also as an antireflection layer. Therefore, the modification of the TiO_2 layer can further enhance the light absorption for our photocathodes.

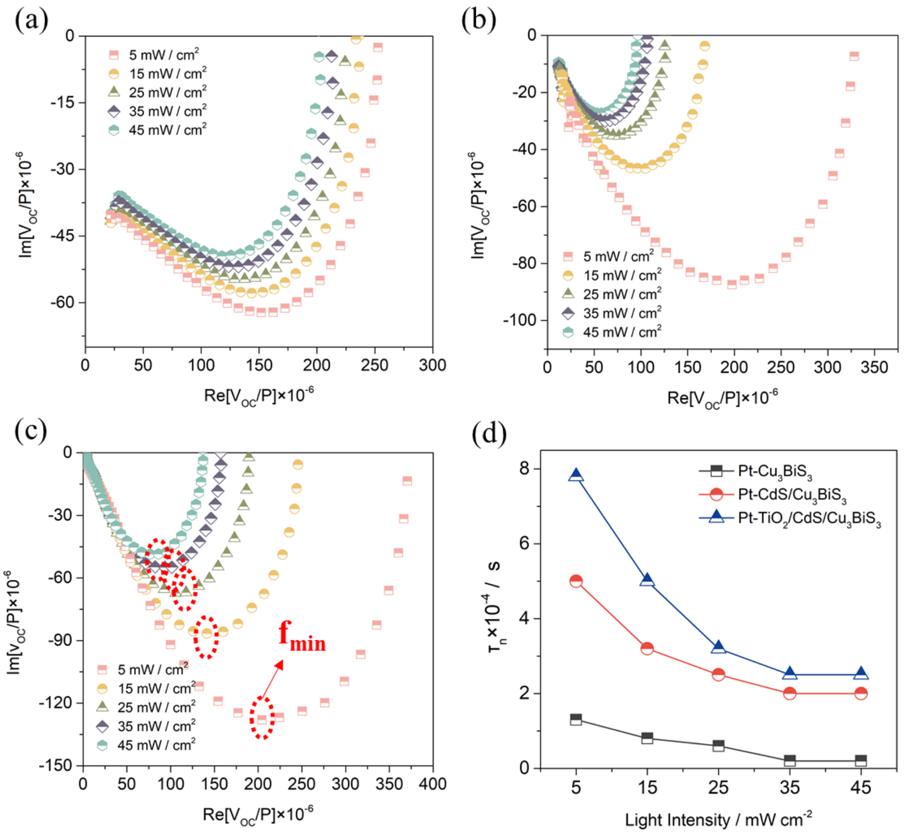

Fig. S10 IMVS spectra of the **a** Pt-Cu_3BiS_3, **b** Pt-CdS/Cu_3BiS_3 and **c** Pt-TiO_2/CdS/Cu_3BiS_3 photocathode at 590 nm in a phosphate buffer solution (pH 6.5); **d** Lifetime of carriers generated by these three photocathodes (The data points is measured in a-c). Source data are provided as a Source Data file.

The IMVS measurements were carried out under 590 nm LED illumination, and the LED light intensity was changed from 5 mW/cm^2 to 45 mW/cm^2. Meanwhile, the IMVS tests were performed under open circuit conditions, indicating that the IMVS test did not involve surface redox reactions and only reflected the transfer of photogenerated carriers inside the electrode.[1,2] As shown in Fig. S10a-c, the lowest point of the semicircular arc was corresponding to the minimum frequency value (f_{min}) in the frequency spectrumthe, and the lifetimes of the photogenerated carriers could be obtained using the following equation:

$$\tau_n = 1/(2\pi f_{min}) \qquad \text{(Equation S1)}$$

The calculated lifetimes values of the three photocathodes were shown in Fig. S9d. It was found that the carriers lifetime of the three electrodes significantly decreased with LED light intensity before 25 mW/cm^2, and remained stable under the high-

intensity LED light (25-45 mW/cm^2). It was attributed to the generation speed of photoexcited carriers and their recombination rate tended to balance and therefore stabilize the carrier lifetime when we increased the LED light intensity.[1,2] In addition, the Pt-TiO_2/CdS/Cu_3BiS_3 photocathode possessed a higher carriers lifetime than that of the Pt-CdS/Cu_3BiS_3 and Pt-CdS/Cu_3BiS_3 photocathode, indicating that the interfacial recombination rate was decreased accompanied with the import of the ALD-TiO_2/CdS overlayer.

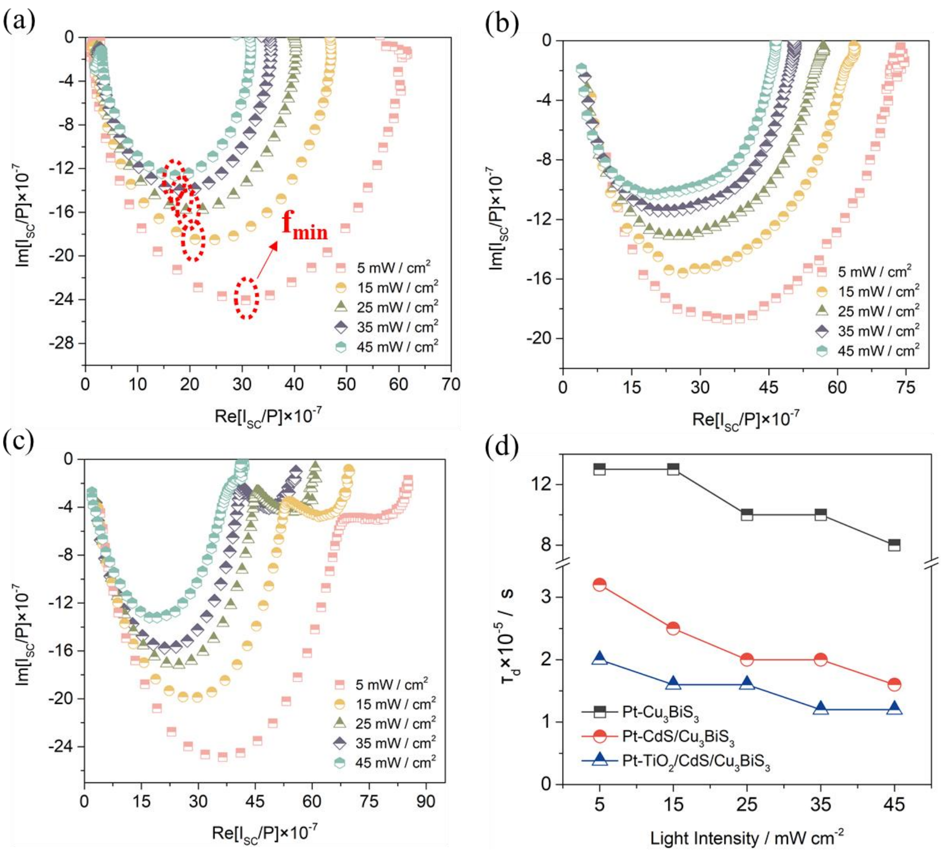

Fig. S11 IMPS spectra of the **a** Pt-Cu_3BiS_3, b Pt-CdS/Cu_3BiS_3 and **c** Pt-TiO_2/CdS/Cu_3BiS_3 photocathode at 590 nm in a phosphate buffer solution (pH 6.5); **d** Transfer time of photogenerated carriers generated by these three photocathodes (The data points is measured in a-c). Source data are provided as a Source Data file.

The IMPS measurements were carried out under 590 nm LED illumination, and the LED light intensity was changed from 5 mW/cm^2 to 45 mW/cm^2. As reported in previous work, IMPS was carried out under short circuit conditions, which could describe the transfer and reaction process of photogenerated carriers within the electrode and reaction at the interface between electrode and electrolyte.[1,2] Fig. S11a-c shows the IMPS Nyquist plots of the three electrodes, the lowest point of the semicircular arc was corresponding to the minimum frequency value (f_{min}) in the frequency spectrumthe, and the transfer time of the photo-induced carriers could be obtained using the following equation:

$$\tau_d=1/(2\pi f_{min}) \qquad \text{(Equation S2)}$$

The calculated transfer time values of the three photocathodes were shown in Fig.

S11d. It can be found that the Pt-TiO_2/CdS/Cu_3BiS_3 photocathode exhibited a lower photo-induced carriers transfer time than that of the Pt-CdS/Cu_3BiS_3 and Pt-CdS/Cu_3BiS_3 photocathode, which indicated that the charge transfer efficiency was effectively improved with the insert of the TiO_2/CdS double layer.[1,2]

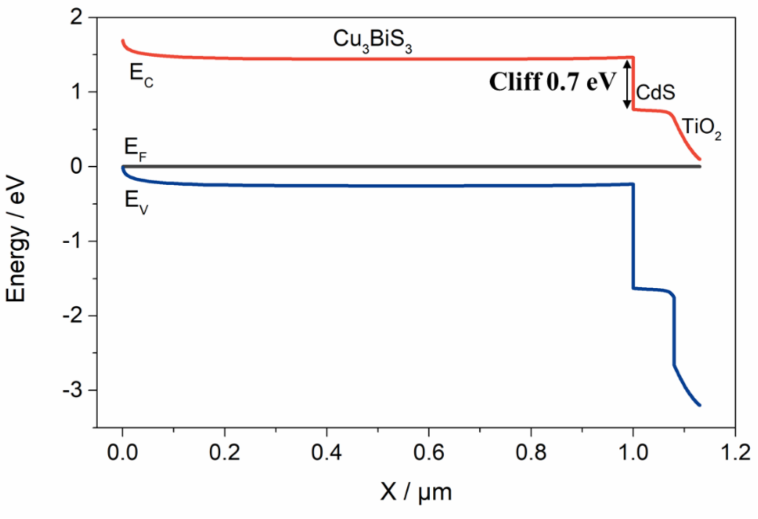

Fig. S12 Band schema (simulated by the SCAPS software) of the $TiO_2/CdS/Cu_3BiS_3$ structure. Source data are provided as a Source Data file.

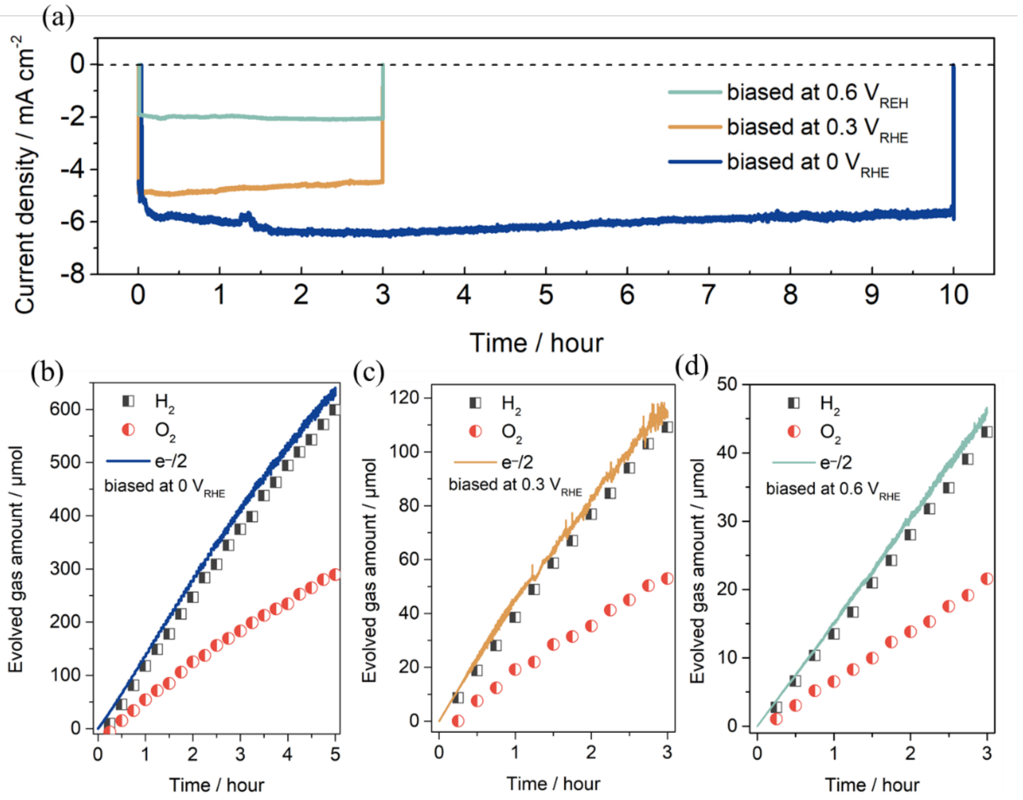

Fig. S13 a Photocurrent density-time curves of Pt-TiO_2/CdS/Cu_3BiS_3 photocathode biased at 0 V_{RHE}, 0.3 V_{RHE} and 0.6 V_{REH}; Hydrogen and oxygen evolution amount from the Pt-TiO_2/CdS/Cu_3BiS_3 photocathode at **b** 0 V_{RHE}, **c** 0.3 V_{RHE} and **d** 0.6 V_{REH} under simulated sunlight irradiation. Source data are provided as a Source Data file.

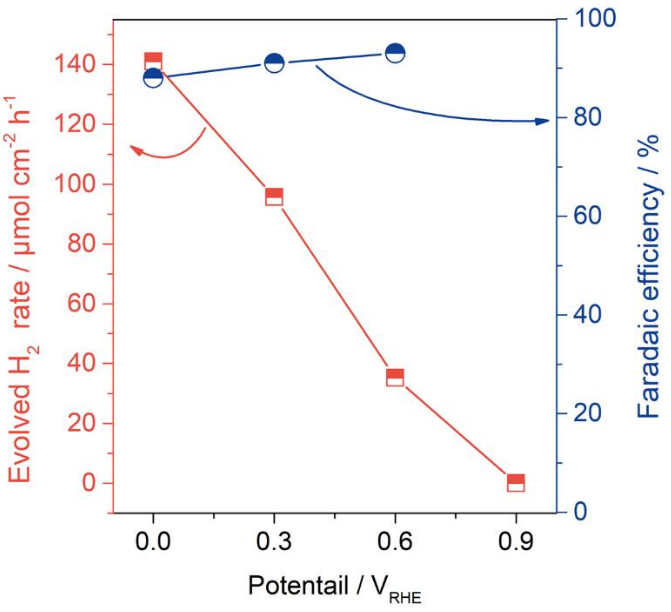

Fig. S14 H_2 gas evolution rate and Faradaic efficiency of the Pt-TiO_2/CdS/Cu_3BiS_3 photocathode biased at various potentials (0 V_{RHE}, 0.3 V_{RHE}, 0.6 V_{RHE} and 0.9 V_{RHE}). Source data are provided as a Source Data file.

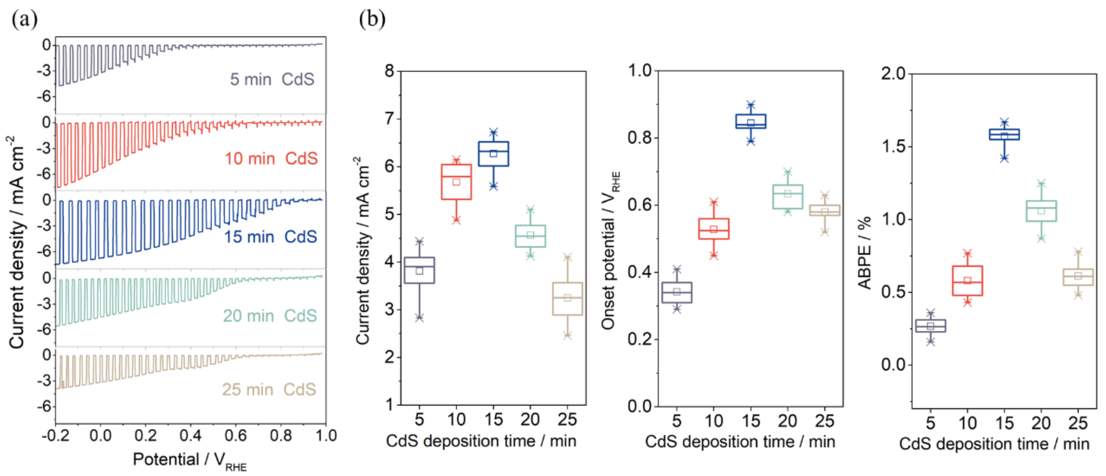

Fig. S15 a The CdS thickness dependent photocurrent density-potential curves of the Cu_3BiS_3-based photocathodes (5 min, 10 min, 15min, 20 min and 25 min for the CBD of CdS layer) and **b** corresponding statistical box data of photoelectrochemical properties (current density, onset potential and ABPE). The box plot denotes median (centre line), mean value (dots), 25th (bottom edge of the box), 75th (top edge of the box), 95th (upper whisker) and 5th (lower whisker) percentiles. The sample size in each column is 10 devices. Source data are provided as a Source Data file.

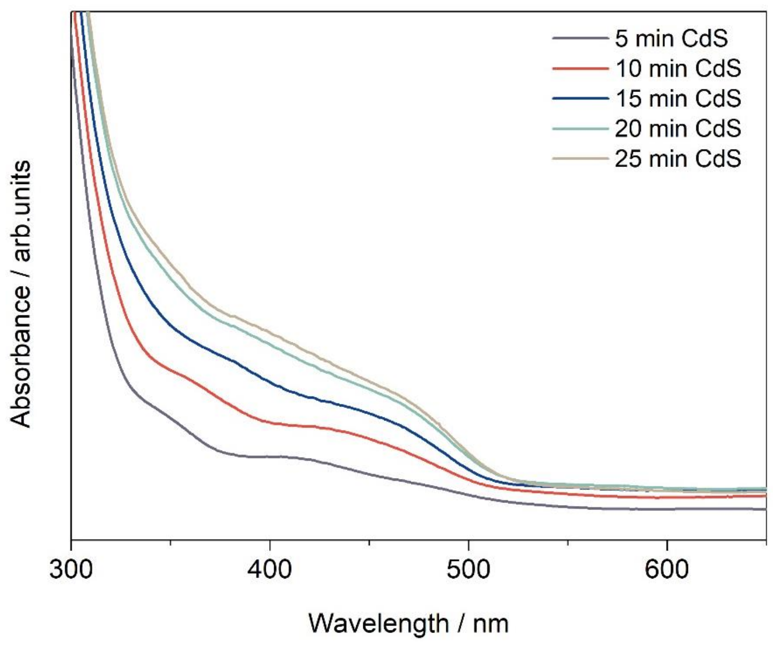

Fig. S16 UV-Vis absorption spectra of the CdS layer with various CBD times. Source data are provided as a Source Data file.

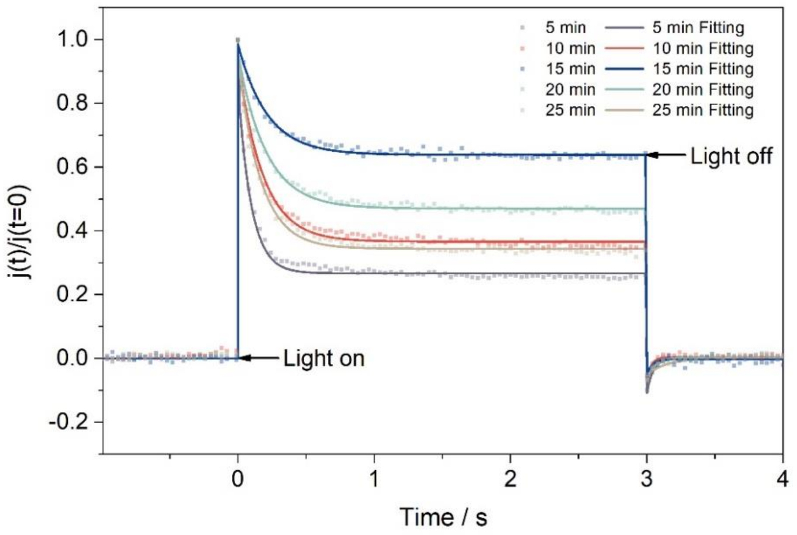

Fig. S17 Transit photocurrent spectrums of the Cu_3BiS_3-based photocathodes modified with different thicknesses of CdS layer (5 min, 10 min, 15min, 20 min and 25 min for the CBD of CdS layer). Source data are provided as a Source Data file.

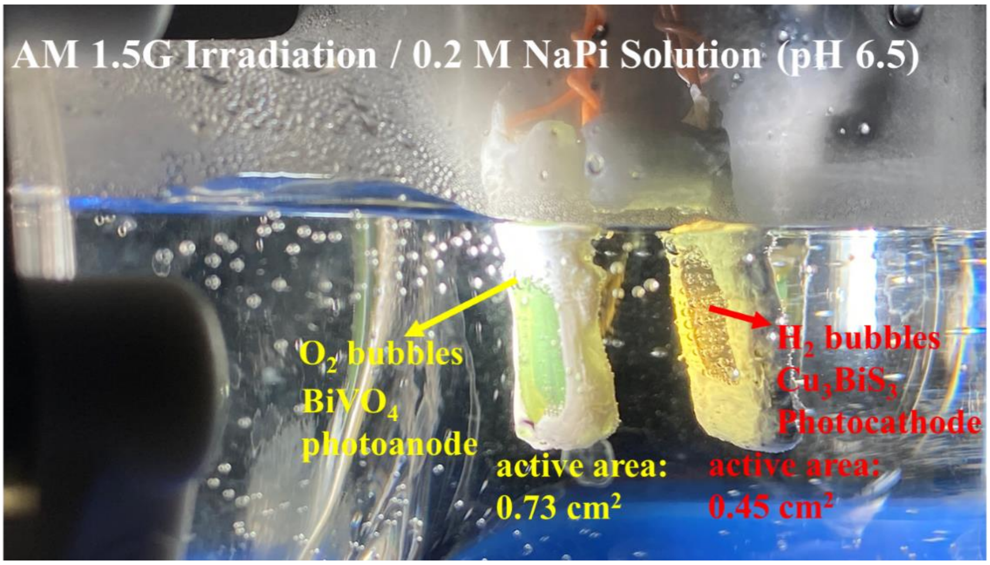

Fig. S18 Visual demonstration of the Cu_3BiS_3-$BiVO_4$ tandem cell under simulated sunlight irradiation.

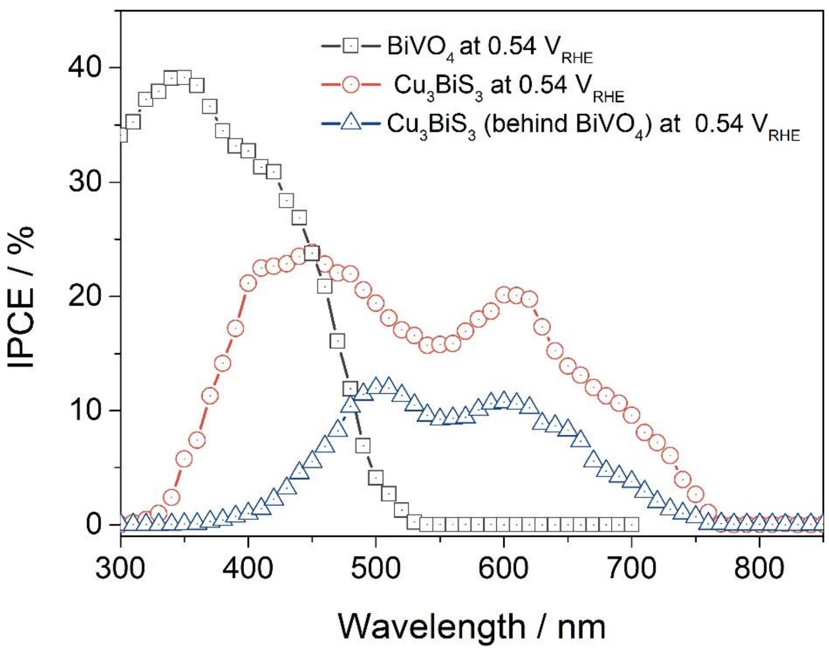

Fig. S19 IPCE spectra (measured under 0.54 V_{RHE}) of the Cu_3BiS_3-based photocathode, $BiVO_4$ photoanode and Cu_3BiS_3-based photocathode behind the $BiVO_4$ photoanode. Source data are provided as a Source Data file.

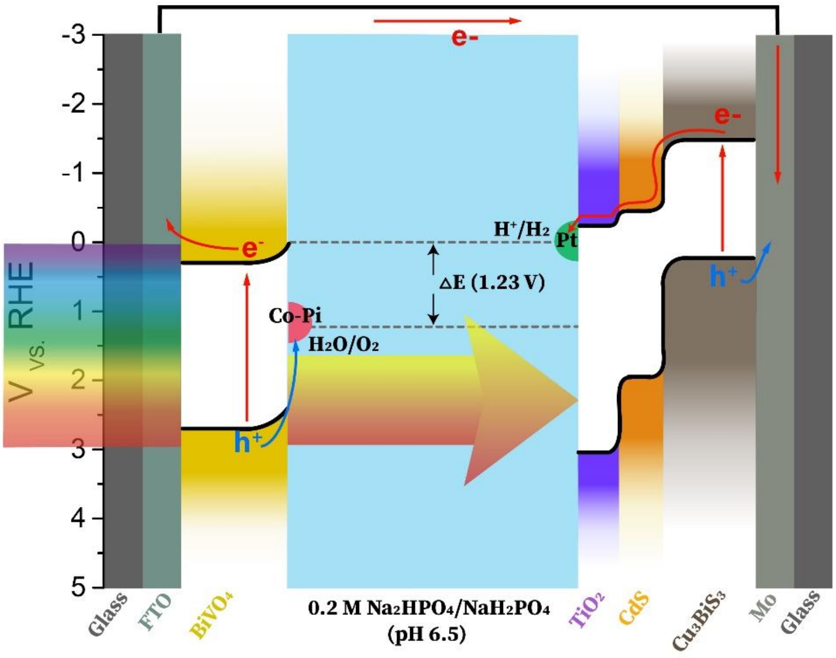

Fig. S20 Energy diagrams of the Cu_3BiS_3-$BiVO_4$ tandem cell for unbiased solar water splitting in pH 6.5 buffer solution.

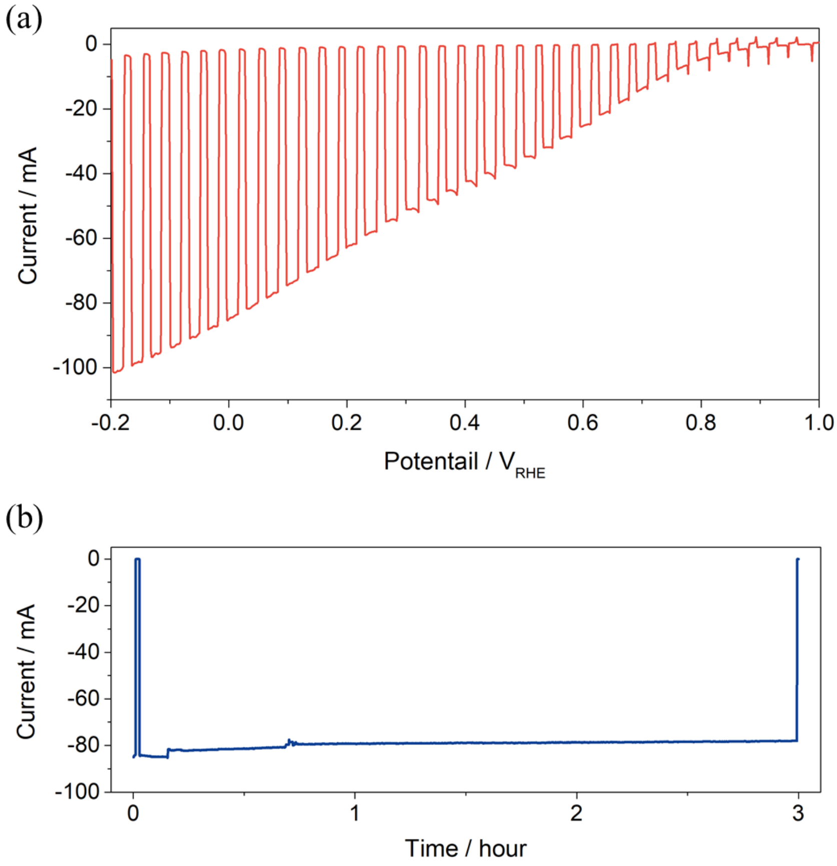

Fig. S21 a Chopped photocurrent-potential curves and **b** photocurrent-time curves of the 5×5 cm^2 size Pt-TiO_2/CdS/Cu_3BiS_3 photocathode under simulated sunlight irradiation (active area: 21 cm^2). Source data are provided as a Source Data file.

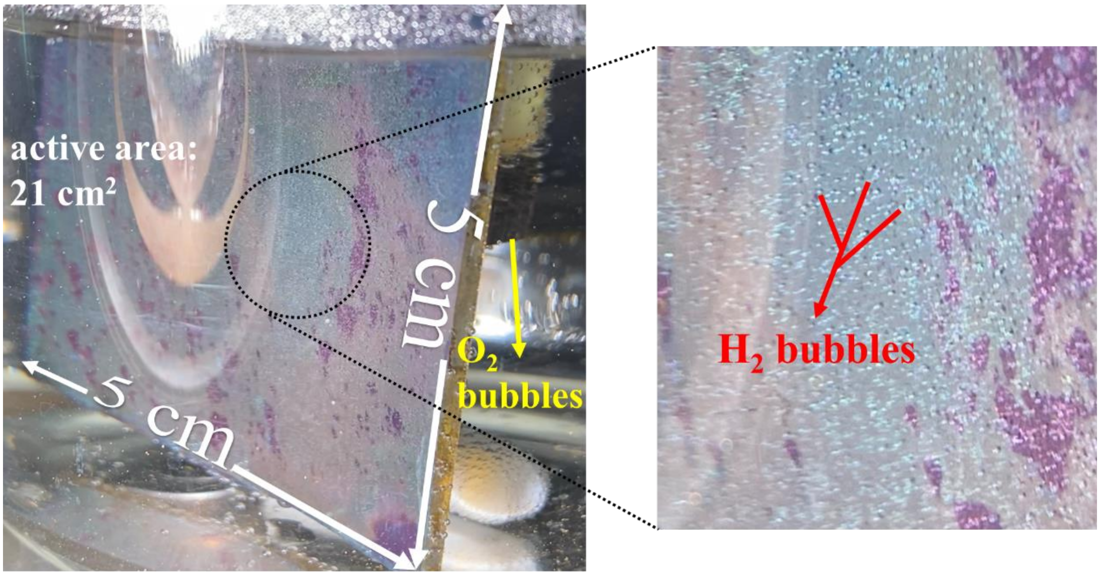

Fig. S22 Photograph of the 5×5 cm^2 size Pt-TiO_2/CdS/Cu_3BiS_3 photocathode (active area: 21 cm^2) at 0 V_{RHE} under simulated sunlight irradiation.

References

1. Zhang, Y.P. et al. Improvement of $BiVO_4$ photoanode performance during water photo-oxidation using rh-doped $SrTiO_3$ perovskite as a co-catalyst. *Adv. Funct. Mater.* **29**, 1902101 (2019).
2. Li, L. et al. Surface plasmon resonance effect of a Pt-nano-particles-modified TiO_2 nanoball overlayer enables a significant enhancement in efficiency to 3.5% for a Cu_2ZnSnS_4-based photocathode used for solar water splitting. *Chem. Eng J.* **396**, 125264 (2020).
