# Supplementary material for: Wittichenite semiconductor of Cu3BiS3 films for efficient hydrogen evolution from solar driven photoelectrochemical water splitting
Source: Nat Commun. 2021 Jun 18;12:3795. doi: 10.1038/s41467-021-24060-5 (PMC8213846; doi:10.1038/s41467-021-24060-5)
Supplement: Supplementary file 6 — Description of additional supplementary files [file 41467_2021_24060_MOESM6_ESM.docx]

Description of additional supplementary information

Title: Supplementary Movie 1

Description: Movie of the laboratory-scale Cu3BiS3-BiVO4 tandem cell under working with simulated sunlight irradiation

Title: Supplementary Movie 2

Description: Movie of the 5×5 cm2 size Pt-TiO2/CdS/Cu3BiS3 photocathode (active area: 21 cm2 ) at 0 VRHE under simulated sunlight irradiation.

Title: Supplementary Movie 3

Description: Movie of the 5×5 cm2 size Cu3BiS3-BiVO4 tandem device under working with simulated sunlight irradiation.
