# Supplementary material for: Wittichenite semiconductor of Cu3BiS3 films for efficient hydrogen evolution from solar driven photoelectrochemical water splitting
Source: Nat Commun. 2021 Jun 18;12:3795. doi: 10.1038/s41467-021-24060-5 (PMC8213846; doi:10.1038/s41467-021-24060-5)
Supplement: Supplementary file 2 — Peer review [file 41467_2021_24060_MOESM2_ESM.pdf]

Reviewers' comments:

Reviewer #1 (Remarks to the Author):

The authors have carried out an impressive, intensive study on optimised Cu_3BiS_3 photocathodes (Voc = 0.9 V vs RHE and J0 = 7mA/cm^2) and their combination with $BiVO_4$ to demonstrate unbiased solar-water splitting with a 2% solar-to-hydrogen efficiency. This device was also fabricated on a 5x5cm scale and photocurrent losses of 30% were recorded over long-term studies of 60h. These is a very interesting result, however, as the authors stated correctly as well, Cu_3BiS_2 photocathodes are not a new invention e.g., J. Yin and J. Jia (CrystEngComm, 2014, 16, 2795) used TiO_2/Cu_3BiS_3 photocathodes already in 2014 and investigated their photoelectrochemical properties. They photoelectrochemical characteristics were not as good as the ones discussed here (Voc = 0.48V and J0 = 6mA), but still close. It is therefore not believed that this manuscript would be of interest to readers of Nat. Commun. There are, moreover, a few aspects in the manuscript which are confusing: (1) The results part begins with a figures from the SI – they should start with discussing figures in the main article. (2) The performance improvement of the Pt-TiO_2/CdS/Cu_3BiS_3 photocathode with respect to the original Pt-Cu_3BiS_3 photocathode seems largely due to the introduction of the protective TiO_2 layer as the authors also state. This suggests that the TiO_2 layer not only has a stabilising effect, but it could potentially also function as an anti-reflection layer and therefore improves the light absorption properties of the device - which is also not novel (see Cheng et al. ACS Energy Lett. 2018, 3, 8, 1795-1800). It is suggested that the authors undertake further absorption/reflection studies of their Pt-TiO_2/CdS/Cu_3BiS_3 device with and without TiO_2 layer. (3) Experimental details are missing, e.g., the acid with which the pH of the buffer solution was adjusted, the photoelectrochemical set-up (light intensity, cell windows etc.), if a two-compartment cell was used for the long-term measurements etc.

Reviewer #2 (Remarks to the Author):

This work reports a Cu_3BiS_3-based photocathode and Cu_3BiS_3-$BiVO_4$ tandem cell for unassisted solar water splitting with remarkable solar-to-hydrogen efficiency (2.04%) and stability (20 h). This manuscript has novelty in that it has created a tandem device with a new photocathode material Cu_3BiS_3. The authors achieved a photocurrent density (7 mA/cm2 at 0 VRHE) 70 times higher than the previously reported peak value (0.1 mA/cm2 at 0 VRHE) for the Cu_3BiS_3 photocathode by applying CdS/TiO_2/Pt. Considering their originality, this reviewer recommends accepting this paper in this journal after only after properly addressing the following concerns by revision, especially Comments 1-2..

1. There appears to be an error in the Mott-Schottky curve for Cu_3BiS_3 in Figure 2b. Since Cu_3BiS_3 is a p-type material, the Mott-Schottky plot should have a negative slope and the flatband potential should be located near the onset potential. However, all curves in Figure 2b have a positive slope with a very negative flat band potential. Why?

2. The authors mentioned there is no obvious secondary phase or impurity phase in Cu_3BiS_3 photocathodes. However, as shown in XRD in Figure 1a and Figure S1, all four Cu_3BiS_3 samples have some impurity such as Copper sulfide or Molybdenum sulfide. Even the optimized sample (380 ℃ annealed) has an impurity peak ($Mo_{2-x}S$). Synthesis should be improved to prepare a pure phase to clearly claim the unprecedented PEC performance of your Cu_3BiS_3-based photocathode. Otherwise, the role of the impurity phase should be clarified with additional material characterization.

3. The notation used throughout the manuscript to indicate the ratio of Cu and Bi seems awkward. [Example - Cu / 3: Bi = 1] They need to be corrected.

4. What is the substrate of the Cu_3BiS_3-based electrode? It is not clearly mentioned.

5. There is a typo. (5*5 cm → 5*5 cm2)

Reviewer #3 (Remarks to the Author):

This paper concerns development of a water-splitting device based on a Cu3BiS3 photocathode and BiVO4 photoanode. The core claim is that Cu3BiS3 will be an industrially competitive solution for high performing, stable devices for solar hydrogen production in the near future.

From the scientific point of view the manuscript is well written and the investigations are detailed and appropriate. Specific comments are given below. However I do feel that the authors have overstated the impact of their work in relation to the state of the art for solar water spltting.

In the introduction, the performance of the Cu3BiS3 device is related to similar device types relying on various photoanode/photocathodes (given in Table 1). In this context, 2% efficiency and 60 hours of stability are reasonable results. But in the context of the state of the art of solar-driven water splitting as a whole, where the theoretical limit of efficiency is around 25% or more, the results are rather modest, and the major challenges in reaching this level are not illuminated. A more balanced introduction is needed so that the reader can put the claims of the authors into proper context. See e.g.

* Energy Environ. Sci., 2015,8, 2811-2824
* Energy Environ. Sci., 2018,11, 1977-1979
* Chem. Soc. Rev., 2019,48, 1908-1971

Throughout the paper, the authors overstate the impact of their results and the status of the field as a whole. e.g.
Page 5: "the Cu3BiS3-based photocathode will attract much attention worldwide and deserves to be widely investigated for large-scale industrial production in the near future."
In fact, the performance of the given device, being far below simple solar-driven water electrolysis, could not possibly make the technology cost effective without major improvements. Similarly, repeated claims of "highly industrialized", "large scale" (meaning 5x5cm), "further industrialization" are all spurious, since there is no industry around this type of device.

This type of language gives a misleading impression to non-specialists, and for specialists it appears that the authors are engaging in false advertising. Therefore it is advisable to moderate the language carefully.

Other comments
* Why were the stability test stopped after short times (20h, 60h) when the devices were still performing quite well? Could data for longer tests be included?
* For the TiO2-protected photocathodes, can the authors comment on the recoverability of performance if a fresh electrolyte is added? What is the cause of current reduction, given that no corrosion was detected?

Replies to Reviewer #1

1) *The authors have carried out an impressive, intensive study on optimised Cu_3BiS_3 photocathodes (Voc = 0.9 V vs RHE and J_0 = 7mA/cm^2) and their combination with $BiVO_4$ to demonstrate unbiased solar-water splitting with a 2% solar-to-hydrogen efficiency. This device was also fabricated on a 5x5cm scale and photocurrent losses of 30% were recorded over long-term studies of 60h. These is a very interesting result, however, as the authors stated correctly as well, Cu_3BiS_3 photocathodes are not a new invention e.g., J. Yin and J. Jia (CrystEngComm, 2014, 16, 2795) used TiO_2/Cu_3BiS_3 photocathodes already in 2014 and investigated their photoelectrochemical properties. They photoelectrochemical characteristics were not as good as the ones discussed here (Voc = 0.48V and J_0 = 6mA), but still close.*

Response:

We thank the reviewer evaluating our work as an impressive, intensive, and very interesting results. We have carefully read the raised paper (CrystEngComm, 2014, 16, 2795) and found that the topic of this paper is different from our submission: J. Jia et al reported Cu_3BiS_3 nanoflower modified TiO_2 nanorod array for the sensitized solar cells, the achieved parameters and efficiency or work conditions of the sensitized solar cells are different from the Cu_3BiS_3 compact film based solar water splitting photoelectrode for hydrogen evolution in our manuscript. The topic of Sensitized solar cell is different from solar water splitting of hydrogen evolution. We thank reviewer introduced the important paper and we have added some introductions regarding on this paper in line 75-77 on page 3 in this revised manuscript.

2) *The results part begins with a figure from the SI – they should start with discussing figures in the main article.*

Response:

We have rewritten the results parts to begin with discussing figures in the main

article, we have re-organized the positions of some discussions in this revised manuscript. (see line 178-193 on page 8 and line 217-221 on page 9.)

3) *The performance improvement of the Pt-TiO_2/CdS/Cu_3BiS_3 photocathode with respect to the original Pt-Cu_3BiS_3 photocathode seems largely due to the introduction of the protective TiO_2 layer as the authors also state. This suggests that the TiO_2 layer not only has a stabilising effect, but it could potentially also function as an anti-reflection layer and therefore improves the light absorption properties of the device - which is also not novel (see Cheng et al. ACS Energy Lett. 2018, 3, 8, 1795-1800). It is suggested that the authors undertake further absorption/reflection studies of their Pt-TiO_2/CdS/Cu_3BiS_3 device with and without TiO_2 layer.*

Response:

According to the valuable suggestion from the reviewer, we have carried out the reflection characterizations of our Cu_3BiS_3-based photocathodes with and without TiO_2 layer. Results were added as **Fig. S9** in this revised SI and brief discussions (line 307-310 on page 13) in our revised main article, and we have copied **Fig. S9** and corresponding discussions as below for your reference.

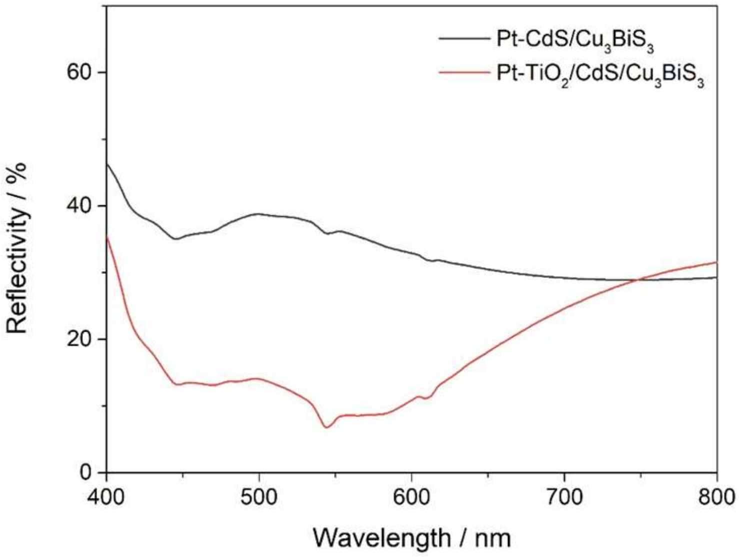

Fig. S9: Reflectivity curves of the Cu_3BiS_3-based photocathodes with and without TiO_2 layer.

In addition, the reflection characterizations (**Fig. S9**) of our Cu_3BiS_3-based photocathodes with and without TiO_2 layer also demonstrated that the TiO_2 layer can be used as an antireflection layer to enhance the light absorption for the Cu_3BiS_3-based photocathodes.

4) *Experimental details are missing, e.g., the acid with which the pH of the buffer solution was adjusted, the photoelectrochemical set-up (light intensity, cell windows etc.), if a two-compartment cell was used for the long-term measurements etc.*

Response:

We have added more experimental details in the photoelectrochemical measurements part of our revised Supporting Information.

<u>Replies to Reviewer #2</u>

This work reports a Cu_3BiS_3-based photocathode and Cu_3BiS_3-$BiVO_4$ tandem cell for unassisted solar water splitting with remarkable solar-to-hydrogen efficiency (2.04%) and stability (20 h). This manuscript has novelty in that it has created a tandem device with a new photocathode material Cu_3BiS_3. The authors achieved a photocurrent density (7 mA/cm^2 at 0 V_{RHE}) 70 times higher than the previously reported peak value (0.1 mA/cm^2 at 0 V_{RHE}) for the Cu_3BiS_3 photocathode by applying CdS/TiO_2/Pt.

Considering their originality, this reviewer recommends accepting this paper in this journal after only after properly addressing the following concerns by revision, especially Comments 1-2：

1) *There appears to be an error in the Mott-Schottky curve for Cu_3BiS_3 in Figure 2b. Since Cu_3BiS_3 is a p-type material, the Mott-Schottky plot should have a negative slope and the flatband potential should be located near the onset potential. However, all curves in Figure 2b have a positive slope with a very negative flat band potential. Why?*

Response:

The Mott-Schottky curves in **Fig. 2b** belong to the finished Cu_3BiS_3 -based photocathodes (Pt-TiO_2/CdS/Cu_3BiS_3). The Moott-Schottky analysis is usually used to study the band bending at the surface of photocathodes that contact to the electrolyte.[1-3] Therefore, the Mott-Schottky plots in **Fig. 2b** showed a positive slope owing to the n-type outer coverage layer-TiO_2. Meanwhile, the photocathode based on the Cu_3BiS_3 film sprayed at 380 possessed most negative Vfb of -0.57 V_{RHE}, which indicated that it was easier for photogenerated electrons to transfer from the electrode into the electrolyte and participate in the hydrogen evolution reaction.[3]

2) *The authors mentioned there is no obvious secondary phase or impurity phase in Cu_3BiS_3 photocathodes. However, as shown in XRD in Figure 1a and Figure S1, all four Cu_3BiS_3 samples have some impurity such as Copper sulfide or Molybdenum sulfide. Even the optimized sample (380 annealed) has an impurity peak ($Mo_{2-x}S$). Synthesis should be improved to prepare a pure phase to clearly claim the unprecedented PEC performance of your Cu3BiS3-based photocathode. Otherwise, the role of the impurity phase should be clarified with additional material characterization.*

Response:

We appreciate the reviewer for the helpful comment. Currently we are difficult to synthesis a completely pure phase Cu_3BiS_3 films in a short time. Based on our knowledge, we should further fine adjustment the spray speed and temperature to achieve a pure phase Cu_3BiS_3 film, detail works are on the way. We do should optimize the synthesis process of the Cu_3BiS_3 films in our future works. Many thanks for your good comments!

3) *The notation used throughout the manuscript to indicate the ratio of Cu and Bi seems awkward. [Example - Cu / 3: Bi = 1] They need to be corrected.*

Response:

The notation of the molar ratio of Cu to Bi has been corrected in the revised manuscript and Supporting Information. For example, we corrected the "Cu/3: Bi=1" as " Cu : Bi=3 : 1".

4) *What is the substrate of the Cu_3BiS_3-based electrode? It is not clearly mentioned.*

Response:

We used the Mo-coated soda-lime glass as the substrate of the Cu_3BiS_3-based electrode. We have added this experimental detail in the preparation of the Cu_3BiS_3 films part of our revised Supporting Information.

5) *There is a typo. (5*5 cm → 5*5 cm^2)*

Response:

Thanks for your helpful suggestion, we have corrected "5×5 cm" as "5×5 cm^2" in this revised manuscript and supporting information.

References

1. Xu, Z.Q., Guan, Z.J., Yang, J.J. & Li, Q.Y. Band positions and photoelectrochemical properties of solution-processed silver-substituted Cu_2ZnSnS_4 photocathode. *ACS Appl. Energy Mater.* **2**, 2779-2785 (2019).
2. Zhou, J., Zhao, J. & Liu, R. Defect engineering of zeolite imidazole framework derived ZnS nanosheets towards enhanced visible light driven photocatalytic hydrogen production. *Appl. Catal. B-Environ.* **278**, 119265 (2020).
3. Yu, X.L. et al. Biomolecule-assisted self-assembly of CdS/MoS_2/graphene hollow spheres as high-efficiency photocatalysts for hydrogen evolution without noble metals. *Appl. Catal. B-Environ.* **182,** 504-512 (2016).

Replies to Reviewer #3

1) *This paper concerns development of a water-splitting device based on a Cu_3BiS_3 photocathode and $BiVO_4$ photoanode. The core claim is that Cu_3BiS_3 will be an industrially competitive solution for high performing, stable devices for solar hydrogen production in the near future. From the scientific point of view the manuscript is well written and the investigations are detailed and appropriate. Specific comments are given below. However I do feel that the authors have*

overstated the impact of their work in relation to the state of the art for solar water spltting.

In the introduction, the performance of the Cu_3BiS_3 device is related to similar device types relying on various photoanode/photocathodes (given in Table 1). In this context, 2% efficiency and 60 hours of stability are reasonable results. But in the context of the state of the art of solar-driven water splitting as a whole, where the theoretical limit of efficiency is around 25% or more, the results are rather modest, and the major challenges in reaching this level are not illuminated. A more balanced introduction is needed so that the reader can put the claims of the authors into proper context. See e.g.

* *Energy Environ. Sci., 2015,8, 2811-2824*
* *Energy Environ. Sci., 2018,11, 1977-1979*
* *Chem. Soc. Rev., 2019,48, 1908-1971*

Throughout the paper, the authors overstate the impact of their results and the status of the field as a whole. e.g.

Page 5: "the Cu_3BiS_3-based photocathode will attract much attention worldwide and deserves to be widely investigated for large-scale industrial production in the near future." In fact, the performance of the given device, being far below simple solar-driven water electrolysis, could not possibly make the technology cost effective without major improvements. Similarly, repeated claims of "highly industrialized", "large scale" (meaning 5x5cm), "further industrialization" are all spurious, since there is no industry around this type of device.

Response:

Reviewer was misunderstood our expression while we only stated that the properties presented from the Cu_3BiS_3-based photoelectrode in this work indicate its great industrial future and will attract much attention worldwide and deserves to be widely investigated for industrial production in future. We have not stated that the Cu_3BiS_3 photoelectrode was already industrialized currently. In order to avoid any misunderstands we have re-written the paragraph from line 98 and line 108-109 on page 4-5 in this revised manuscript.

2) *Why were the stability test stopped after short times (20h, 60h) when the devices were still performing quite well? Could data for longer tests be included?*

Response:

We have added the stability performances for another more 10 hours and 20 hours after the 20-hours (**Fig. 4b**) and 60-hours (**Fig. 4g**) stability test as below, respectively:

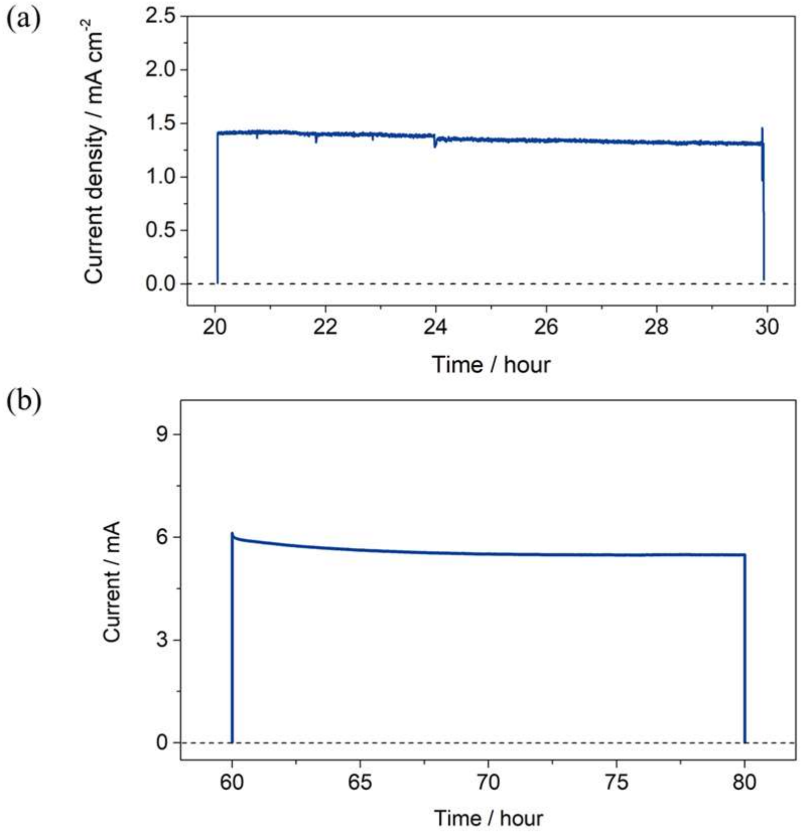

Fig. R1: Later 10-hour (a) and 20-hour (b) current-time curves after the 20-hours (**Fig. 4b**) and 60-hours (**Fig. 4g**) stability test.

As shown in **Fig. R1a** and **b**, the photocurrents of the tandem cells were degraded in trend.

3) *For the TiO_2-protected photocathodes, can the authors comment on the recoverability of performance if a fresh electrolyte is added? What is the cause of current reduction, given that no corrosion was detected?*

Response:

Thanks for the reviewer's helpful comment. According to our experimental experience, the PEC performance of our Cu_3BiS_3-based photocathode can partial recover after adding a fresh electrolyte. In fact, TiO_2 layer is not an excellent protective layer to completely prevent photocorrosion of the photocathode. The photocurrent of many types of photocathodes that protected by the TiO_2 layer usually exhibited a different degrees of decrease during their stability test.[1-4] Therefore, the electrolyte that continuously heated up by the simulated sunlight irradiation can get cooled down after adding a fresh electrolyte, which can slow down the photocorrosion of CdS layer and further slowdown the process of photocurrent drop. On the other hand, the concentration of electrolyte can also be supplemented after adding a fresh electrolyte.

Furthermore, we found that the photocurrent of the Cu_3BiS_3-based photocathode would be enhanced after a new round of Pt catalyst deposition. This phenomenon also be found by Jooho Moon et al.,[5] they thought that the detachment of Pt particles due to the releasing of large bubbles is one of the well-known degradation mechanisms in the Pt-decorated photocathodes for water splitting, and it is widely reported in the related literature.[6]

References

1. Yang, W. et al. Molecular chemistry-controlled hybrid ink derived efficient Cu_2ZnSnS_4 photocathodes for photoelectrochemical water splitting. *ACS Energy Lett.* **1**, 1127-1136 (2016).
2. Zhou, Y.H. et al. Efficient and stable $Pt/TiO_2/CdS/Cu_2BaSn(S,Se)_4$ photocathode for water electrolysis applications. *ACS Energy Lett.* **3**, 177-183 (2018).
3. Zhang, L. et al. Scalable low-band-gap Sb_2Se_3 thin-film photocathodes for efficient visible-near-infrared solar hydrogen evolution. *ACS Nano* **11**, 12753-12763 (2017).
4. Chen, M.X. et al. Spatial control of cocatalysts and elimination of interfacial defects towards efficient and robust CIGS photocathodes for solar water splitting. *Energy Environ. Sci.* **11**, 2025-2034 (2018).
5. Yang, W. et al. Benchmark performance of low-cost Sb_2Se_3 photocathodes for unassisted solar overall water splitting. *Nat. Commun.* **11**, 861 (2020).
6. Yang, W., Prabhakar, R.R., Tan, J., Tilley, S.D. & Moon, J. Strategies for enhancing the photocurrent, photovoltage, and stability of photoelectrodes for photoelectrochemical water splitting. *Chem. Soc. Rev.* **48**, 4979-5015 (2019).

REVIEWERS' COMMENTS

Reviewer #2 (Remarks to the Author):

I am fully satisfied with the revision with response to my comments, and now recommend its acceptance for puiblication in the current form.
